# Analysis of the Ordering Effects in Anthraquinone Thin Films and Its Potential Application for Sodium Ion Batteries

**DOI:** 10.1021/acs.jpcc.0c10778

**Published:** 2021-02-10

**Authors:** Daniel Werner, Dogukan H. Apaydin, Dominik Wielend, Katharina Geistlinger, Wahyu D. Saputri, Ulrich J. Griesser, Emil Dražević, Thomas S. Hofer, Engelbert Portenkirchner

**Affiliations:** † Institute of Physical Chemistry, University of Innsbruck, 6020 Innsbruck, Austria; ‡ Institute of Materials Chemistry, TU Wien, 1060 Vienna, Austria; § Linz Institute for Organic Solar Cell (LIOS), Institute of Physical Chemistry, Johannes Kepler University Linz, 4040 Linz, Austria; ∥ Institut für Ionenphysik und Angewandte Physik, Universität Innsbruck, 6020 Innsbruck, Austria; ⊥ Austrian-Indonesian Centre (AIC) for Computational Chemistry, Universitas Gadjah Mada, Sekip Utara, Yogyakarta 55281, Indonesia; # Indonesian Institute of Sciences, Sasana Widya Sarwono (SWS), 12710 Jakarta, Indonesia; ∇ Institute of Pharmacy, University of Innsbruck, 6020 Innsbruck, Austria; ○ Department of Biological and Chemical Engineering, Aarhus University, 8200 Aarhus N, Denmark; ◆ Theoretical Chemistry Division, Institute for General, Inorganic and Theoretical Chemistry, University of Innsbruck, 6020 Innsbruck, Austria

## Abstract

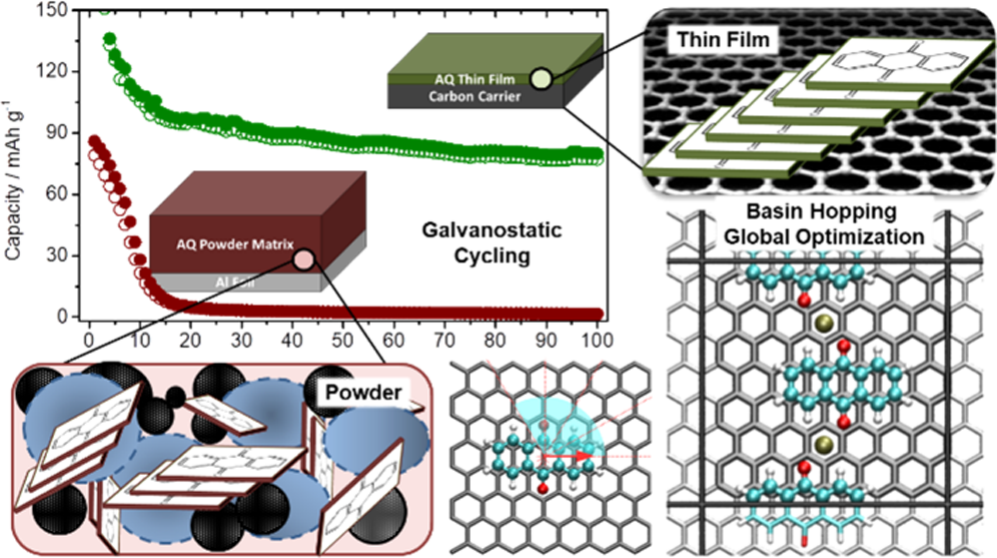

The ordering effects in anthraquinone (AQ) stacking forced by thin-film application and its influence on dimer solubility and current collector adhesion are investigated. The structural characteristics of AQ and its chemical environment are found to have a substantial influence on its electrochemical performance. Computational investigation for different charged states of AQ on a carbon substrate obtained via basin hopping global minimization provides important insights into the physicochemical thin-film properties. The results reveal the ideal stacking configurations of the individual AQ-carrier systems and show ordering effects in a periodic supercell environment. The latter reveals the transition from intermolecular hydrogen bonding toward the formation of salt bridges between the reduced AQ units and a stabilizing effect upon the dimerlike rearrangement, while the strong surface–molecular interactions in the thin-film geometries are found to be crucial for the formed dimers to remain electronically active. Both characteristics, the improved current collector adhesion and the stabilization due to dimerization, are mutual benefits of thin-film electrodes over powder-based systems. This hypothesis has been further investigated for its potential application in sodium ion batteries. Our results show that AQ thin-film electrodes exhibit significantly better specific capacities (233 vs 87 mAh g^–1^ in the first cycle), Coulombic efficiencies, and long-term cycling performance (80 vs 4 mAh g^–1^ after 100 cycles) over the AQ powder electrodes. By augmenting the experimental findings via computational investigations, we are able to suggest design strategies that may foster the performance of industrially desirable powder-based electrode materials.

## 1 Introduction

Organic semiconducting materials based on small molecules like quinones, which build the core semiconductor element, have gained increasing interest as novel candidates for nature-derived organic electrodes.^1^ Such molecules are found in the bark and roots of certain plants and have previously been utilized as centuries-old natural dyes.^2^ More recently, other useful commercial applications, such as organic field-effect transistors (OFETs), organic light-emitting diodes (OLEDs), and organic solar cells (OPVs), have been added, owing to their high conductivity and minimum footprint in nature.^3−6^ These low-molecular-weight carbonyl compounds are processable into thin films that demonstrate a long-range order due to extensive intermolecular π–π interactions, resulting in a decent charge carrier transport and current collector adhesion.^1,7−9^ Additionally, carbonyl compounds are also capable of reversible charge storage via an enolization-type reduction reaction and a reverse oxidation reaction of the carbonyl group, leading to energy densities and power-rate performances that are comparable or superior to state-of-the-art lithium (Li) ion batteries (LIBs).^6,10−12^ Especially anthraquinone (AQ) and its derivatives^13^ have been previously investigated as promising cathode materials for various organic metal ion batteries,^5,14−16^ due to their high theoretical capacity of 257 mAh g^–1^.^17−24^ Since their redox potential fits well within the voltage stability window of most battery electrolytes, AQ-based electrodes may also serve as model compounds for the study of low-cost and energy-efficient electrodes. In this regard, the increasing demand for portable, high-power, and low-cost energy storage has triggered substantial scientific interest to find alternatives to the resource-limited Li-ion system. Consequently, the research on sodium (Na) ion batteries (SIBs) has lately been intensified.^25^ The choice for Na as the central metal ion is mainly driven by its high natural abundance, being about ten thousand times higher than that for Li.^26−28^ In recent years, increasing efforts have been made toward the development of high-capacity materials for Na ion batteries,^29,30^ especially for organic materials that are inherently eco-efficient and environmentally friendly.^14^ At the same time, progressive engineering efforts resulted in feasible approaches toward low-cost production and recyclability.^31^

Major issues that have hindered the utilization of organic molecules such as AQ in battery applications so far are the high solubility in polar organic battery electrolytes (i.e., ethylene carbonate (EC) and dimethyl carbonate (DMC)) and chemical degradation, resulting in poor cycle-life performance.^11,12,32,33^ On the other hand, departing from the concept of small molecules, the polyanionic AQ cathodes recently proposed are more stable but allow only for much smaller theoretical capacities.^34,35^ Prior research showed that AQ pigment molecules are found to associate to form dimers, trimers, and other polyaggregates.^36^ Intermolecular dimerization decreases the available Na ion storage sites, resulting in a deviation from the ideal storage capacity of two electrons per molecule and hence adding to the chemical degradation issue. On the other hand, dimerization and oligomerization have been proven to be beneficial to suppress the dissolution of the organic active materials, resulting in an extended cycle-life performance, if the formed dimers remain to be redox-active.^37^ The latter has significant implications for the scale-up capability of organic small molecules in battery applications. Although it is found that AQ and other pigment molecules in general (hydrogen-bonded molecules in particular) tend to be assembled in very ordered and strongly bound crystalline structures in their pure, pristine form, their cycle-life is found to decrease significantly when applied in a powder-based electrode matrix. Such powder-based systems consist of carbon additives (such as carbon black) and binders mixed with the active material, as typically used in the process of battery electrode preparation in pursuit of an industrially viable application.^38^ While the latter may be an important step toward the scale-up capability of battery electrode preparation, we show that this leads to a significant decrease in the cycle-life performance of organic electrodes that are based on low-molecular-weight compounds as the active storage material.

In this work, we investigate the mechanistic ordering in AQ stacking forced by thin-film application and its effect on dimer solubility and current collector adhesion. Highly ordered, thermally evaporated, thin films of AQ on a carbon carrier substrate are compared to AQ electrodes employing a powder-based system. While the latter is closer to the industry, the AQ thin-film approach reveals significant, substrate-dependent dimerization accompanied by good current collector adhesion, with mutual benefits toward its potential application in SIBs. In addition, computational investigations for different forms of AQ on the carbon carrier are performed, providing manifold insight into the physicochemical properties of these highly complex systems at the molecular level.

## 2 Methods

### 2.1 Electrode Preparation

The AQ thin-film electrodes were prepared by thermal evaporation of the active AQ molecules on top of the carbon carrier substrate. Therefore, carbon carrier discs with a diameter of 17 mm were punched out of a carbon sheet (MGL370, thickness: 0.3 mm). Commercially available AQ (97% purity from Sigma Aldrich) was initially further purified by sublimation using a tube furnace at 250 °C for 20 h. Afterward, the purified AQ was placed in a self-made thermal evaporator. The 200 nm thick AQ films were prepared by subliming AQ at 57 °C at a pressure of 10^–6^ mbar, resulting in an AQ areal loading of approx. 28 μg cm^–2^. The AQ powder electrodes have the following composition: 75% AQ (Sigma Aldrich 97%, used without further purification), 15% binder (PVDF Binder for Li-ion Battery Electrodes, 99%, MTI Corporation), and 10% active carbon (conductive acetylene black for Li-ion battery, 35–45 nm particle size, MTI Corporation, batch number 130502). First, AQ was mortared with acetylene black to obtain a homogeneous mixture of powders. Then, a certain amount of 4% PVDF solution in NMP (*N*-methyl-2-pyrrolidone, 99.5%, VWR) was added to form an inklike slurry where the ratio of AQ/binder/acetylene black was 75:10:15. Films of 10 μm thickness were thereafter coated on an aluminum foil, which served as a mechanical support and the current collector, resulting in an AQ areal loading of approx. 1.12 mg cm^–2^. Films were coated using a doctor-blade machine (Automatic Film Coater with 12″W × 24″L Vacuum Chuck and 250 mm adjustable doctor blade, MTI Corporation).

### 2.2 Electrode Morphology and Spectroscopy

Scanning electron micrographs (JEOL JSM-7601F field-emission electron microscope) were acquired with an electron acceleration voltage of 7 kV using the secondary electron detector. Infrared spectra (diamond ATR, PIKE GaldiATR, Bruker Vertex 70) were measured in the range of 4000–400 cm^–1^ with a resolution of 2 cm^–1^ (32 scans per spectrum).

### 2.3 Battery Assembling and Electrochemical Measurements

The electrochemical measurements were carried out in a three-electrode ECC-Ref Cell (El-Cell) using a Biologic VMP3 potentiostat at room temperature. Sodium metal (Na rod in paraffin oil, VWR, 99.5%) was used as the counter and the reference electrodes and a glass fiber disc (Ø = 18 mm, thickness 1.55 mm, El-Cell) as the separator. The electrolyte (Solvonic, 99%) used was 1 M NaFSI (sodium bis(fluorosulfonyl)imide) in a 1:1 (v/v) mixture of ethylene carbonate (EC) and dimethyl carbonate (DMC). The cells were produced in an Ar-filled glovebox (UNI-lab, MBraun) with the water and oxygen contents below 0.1 ppm. All potentials are reported vs the Na/Na^+^ reference potential.

### 2.4 Theoretical Calculations

To investigate the ideal conformations of AQ, AQ-Na, and AQ-Na_2_ and the respective dimers, theoretical calculations of the associated surface motifs on a carbon model system (i.e., graphite) were carried out. With the aim of keeping the computational effort manageable, the increasingly successful self-consistent charge density functional tight-binding (SCC DFTB) formalism was employed,^39,40^ in conjunction with the 3ob parameter set^41,42^ and the application of the D3 dispersion correction,^43,44^ using the program DFTBPLUS.^45^ This enabled the treatment of comparably large systems consisting of up to six layers of carbon, each containing 112 carbon atoms (i.e., 448 valence electrons per layer). Data visualization of all structures was carried out using the program VMD.^46^

To achieve an adequate compromise in system size and execution time, a 7 × 8 supercell of graphene (56 C_2_ units, 112 atoms) was constructed. Owing to the adsorbed active molecules, the hexagonal symmetry of the surface structure cannot be exploited in the calculations; therefore, the cell was transformed into a Euclidean geometry with the new axes labeled *a* and *b*′ (see Supporting Information, Figure S1). The latter are arranged along the *x*- and *y-*axis of the coordinate frame, respectively, while the *z*-axis represents the nonperiodic direction. To achieve 2D periodicity in the DFTB calculation, the unit cell parameter *c* along the *z*-direction was set to 50 nm.

As shown in the Results and Discussion section, this particular choice of the unit cell is beneficial when studying the adsorption properties of the oxidized forms AQ-Na and AQ-Na_2_ while at the same time ensuring that the surface model remains close to a square, the latter being beneficial to ensure similar sampling along the reciprocal directions, 1/*a* and 1/*b*′. In the next step, this initial structure was subjected to energy minimization considering both the atomic positions as well as the 2D lattice constants while keeping the respective angle fixed. The associated minimum structure yields a unit cell of 1.753 × 1.735 nm. This minimum structure corresponds to a unit cell size *a*_graph_ of 0.2504 nm per C_2_ unit, being approx. 1.8% larger than the reported unit cell parameter of graphene of 0.246 nm.^47^

Next, a total of 85 individual energy minimizations per AQ monomer were carried out, each starting from a different initial structure, thereby considering 13 angular increments in the range [0, 120°] in conjunction with five increments along the *a*-axis in the range of [0, *a*_graph_/2] (see the sketch in the Supporting Information, Figure S1). This optimization strategy, known as basin hopping,^48^ enables a discretization of the underlying potential energy surfaces (PESs) with the aim of identifying the energetically best binding motif of the active molecules on the surface. In this approach, several local energy minimizations from different starting structures are carried out with the aim of transforming individual basins of attraction on the potential energy landscape into a collection of interpenetrating staircases.^48^ This provides an efficient strategy to locate the global minimum, which in the present work corresponds to the ideal binding motif of the substrates on the carrier surface.

This procedure was repeated for surface models containing two, three, and four layers of the carrier material (i.e., systems containing 224, 336, and 448 carbon atoms corresponding to 896, 1344, and 1792 valence electrons) to monitor the convergence properties in the binding energy. It is important to note that all atoms of the system including those of the carbon carrier were included in the structure optimization, i.e., the interlayer separation was optimized for the different systems as well. The best motifs identified for the four-layer system were further reoptimized considering five and six layers of the carrier material corresponding to surface systems containing 570 and 672 carbon atoms (i.e., 2280 and 2688 valence electrons in addition to those contributed by the AQ molecules). The associated binding potential of the surface–molecular interaction *U*_int_ is then estimated as
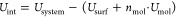
1with *U*_system_ being the total energy obtained for a specific AQ-carrier system and *U*_surf_ and *U*_mol_ being the energies of the isolated *n*-layer surface model and the *n*_mol_ molecular species obtained at the respective minimum AQ structures within the same periodic calculation setup.

To identify the best conformation of the associated dimers (i.e., AQ_2_, AQ_2_-Na_2_, and AQ_2_-Na_4_), a second unit of the active molecules was arranged in the surroundings of an ideal binding motif identified in the previous step. In the case of the dimer structures, *U*_int_ not only accounts for the surface–molecular interactions but also for the molecular–molecular interactions. However, when comparing the energies of the isolated monomers with those of the isolated dimers (i.e., energy minimization carried out in the periodic unit cell but in the absence of the substrate carrier), *U*_int_ can be further decomposed into contributions arising from the surface–molecular and the molecular–molecular interactions.

To obtain an estimate of the minimum distance between the active molecules and the surface, the average *z*-coordinate of all atoms in the uppermost layer of the carrier substrate was determined, while in the case of the active molecules, the average *z*-coordinate of a group of atoms was evaluated (e.g., all carbon atoms of AQ). The averaged atom–surface distance is then obtained as the difference between the respective averages.

To assess the performance of the SCC DFTB/3ob method, calculations of a single anthraquinone molecule on a graphene layer were carried out using 2D-periodic GGA and hybrid density functional theory (DFT) as implemented in the CRYSTAL17 program.^49^ The functionals PBESOL,^50^ LC-wPBESOL,^51^ HSESOL,^52^ and B3LYP^53^ were employed in conjunction with the newly revised triple zeta valence polarization (TZVP) basis sets reported by Peintinger and co-workers.^54,55^ Due to the dramatically increased computational demand of DFT methods compared to their DFTB counterpart, calculations considering only a single layer of the carrier material proved feasible.

## 3 Results and Discussion

### 3.1 Electrode Morphology and Spectroscopy

The morphology of the AQ thin-film and AQ powder-based electrodes is investigated by scanning electron micrographs (SEM, Figure S2). The AQ thin-film electrodes are characterized via a highly intertwined and interconnected network of approx. 10 μm thick fibers, determined by the carbon carrier substrate (Figure S2a,c). The deposited AQ material (approx. 200 nm mean thickness) covers the subjacent carbon fibers of the carrier substrate as a thin, acicular film. Differently, the morphology of the AQ powder-based electrodes is given by a relatively homogeneous network of microparticles forming a connected yet porous network of approx. 10 μm thick film (Figure S2b,d).

**Figure 1:**
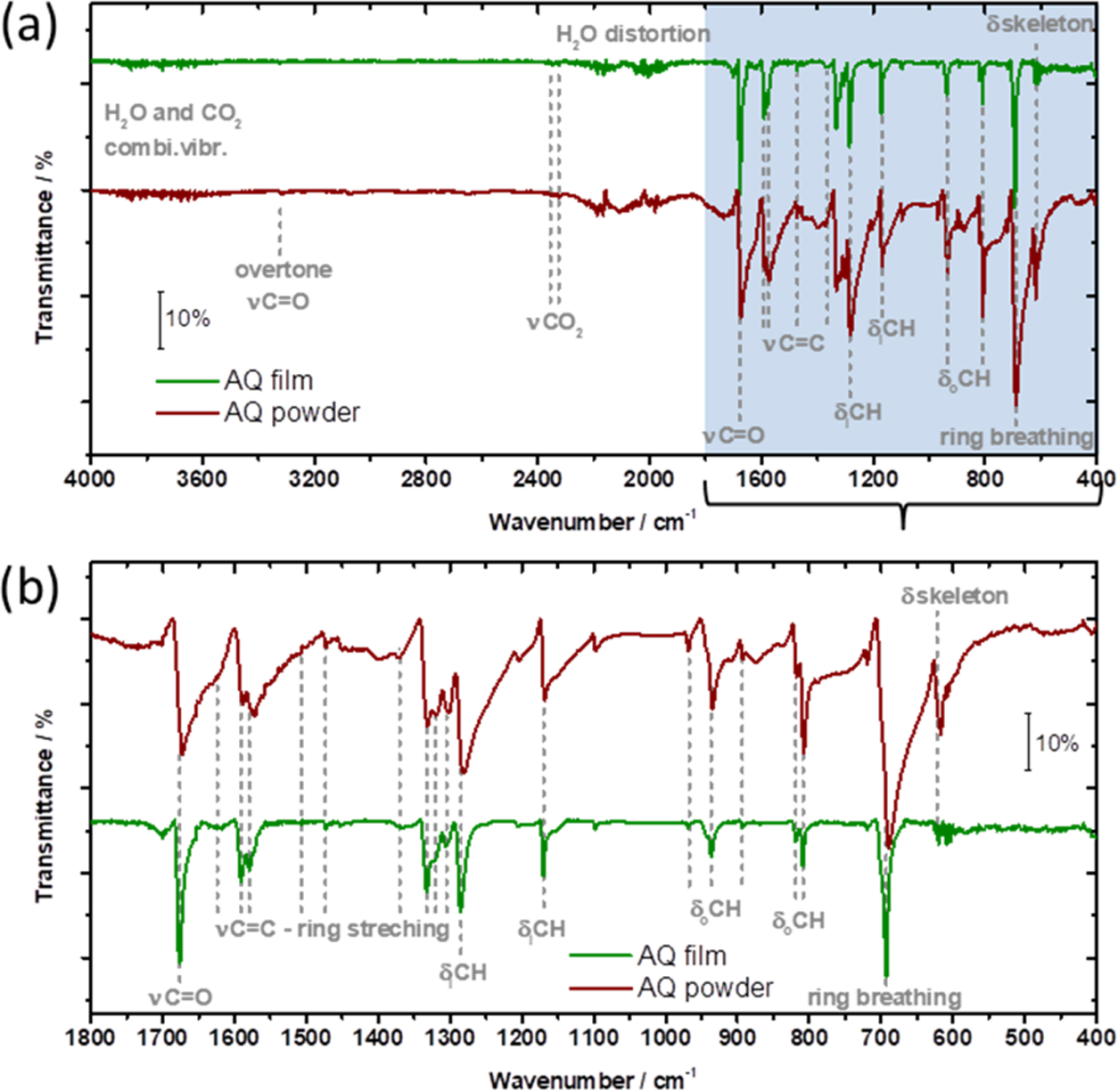
ATR-FTIR spectra of AQ thin-film (green line) and AQ powder (red line) electrodes in the frequency range from (a) 4000 to 400 cm^–1^ and (b) 1800 to 400 cm^–1^.

The vibrational modes of the AQ thin-film and AQ powder electrodes were investigated by ex situ attenuated total reflection (ATR) Fourier transform infrared (FTIR) spectroscopy (Figure 1). A spectrum from 4000 to 400 cm^–1^ is shown in Figure 1a, and the magnified comparison of the low-wavenumber region, from 1800 to 400 cm^–1^, is shown in Figure 1b. An ATR-FTIR spectrum of the pure carbon carrier substrate is given in the Supporting Information, Figure S3 (black line). In general, the signal at around 1670 cm^–1^ is most characteristic for the AQ active material, originating from the stretching vibrations of the carbonyl groups (ν C=O) in quinones that are sometimes characterized by a splitting, probably due to a Fermi resonance. The ATR-FTIR spectra show intense bands at 1625, 1590, 1579, 1507, 1474, and 1366 cm^–1^, which, together with the three bands at 1333, 1322, and 1307 cm^–1^, belong to the ν C=C and ring stretching vibrations of the active AQ material.^56−60^ The absorption signals at 1285 and 1170 cm^–1^ are characteristic of the δ_i_CH vibrations of AQ. The region below 1000 cm^–1^ shows absorptions due to CH out-of-plane bending (δ_o_) vibrations at 969, 936, 893, 819, and 809 cm^–1^. The intense peak at 693 cm^–1^ is associated with the characteristic ring breathing of AQ.^57^ Skeleton deformation (δ skeleton) vibrations show an absorption signal in the CO_2_ distortion area at 620 cm^–1^.^57−59^

In comparison, the reference ATR-FTIR spectrum of the pure carbon carrier substrate (Supporting Information, Figure S3, black line) shows two bands with low intensity at 2916 and 2850 cm^–1^, corresponding to the antisymmetric and symmetric stretching vibrations (ν_as_ and ν_s_) of aliphatic CH_2_ of the carbon carrier substrate. Additional bands with even lower intensities from 1580 to 1500 cm^–1^ and at 1459 cm^–1^ are assigned to ν C=C and CH_2_ bending (δ) vibrations of the carbon carrier substrate, respectively. The noisy signal in the spectra at around 3700 and 1900 cm^–1^ is characteristic for adsorbed water vibrations in combination with distortion vibrations of water vapor in the gas phase. Atmospheric CO_2_ shows a two bands at 2360 and 2330 cm^–1^ (stretching vibrations) and a band at ∼600 cm^–1^ (distortion vibrations) and contributes to the noisy signal at around 3700 cm^–1^ (combination vibrations).^61^

**Figure 2:**
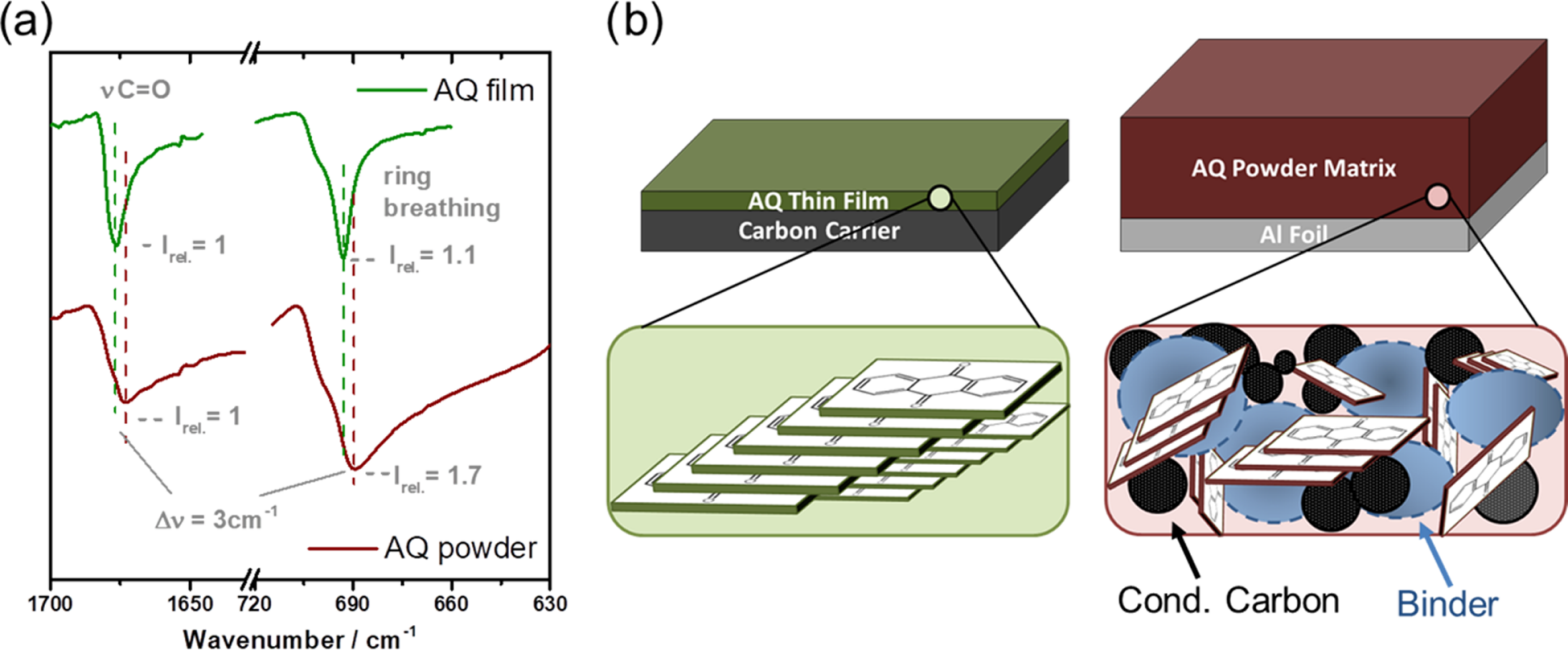
(a) Comparison of the carbonyl stretching (ν C=O) and ring breathing vibrations of AQ thin-film (green line) and AQ powder (red line) electrodes. The comparison shows a shift in the band positions by 3 cm^–1^ to lower wavenumbers for the powder-based electrode. (b) Schematic illustration of AQ thin-film compared to the AQ powder electrodes with conductive carbon and binder additives.

All IR signals of AQ thin-film and AQ powder electrodes are identical in wavenumbers, except the carbonyl stretching (ν C=O) and ring breathing vibrations. These signals are characterized by a small red-shift of three wavenumbers for AQ thin-film electrodes, as shown in Figure 2a. This shift can be explained due to stronger π–π interactions between AQ molecules and AQ with the carbon carrier substrate in the highly ordered thin-film electrodes, as compared to AQ in the powder matrix, where conductive carbon and binder additives hinder long-range π–π stacking (Figure 2b). The π–π interactions reduce the electron density in the aromatic rings, similar to an electron-withdrawing group, which strengthens the C=O bond and shifts the absorption to higher wavenumbers.^61^ While the ratio between the intensities of ν C=O and all other bands in the spectra are similar, the ratio of the ring breathing absorptions differs significantly (Figure 2a), being 1.1 for the AQ thin-film compared to 1.7 for the AQ powder electrode (normalized to the ν C=O intensity). This ratio is known to increase with disorder and is higher for powder than for highly crystallized films.^58^

### 3.2 Electrochemical Characterization

**Figure 3:**
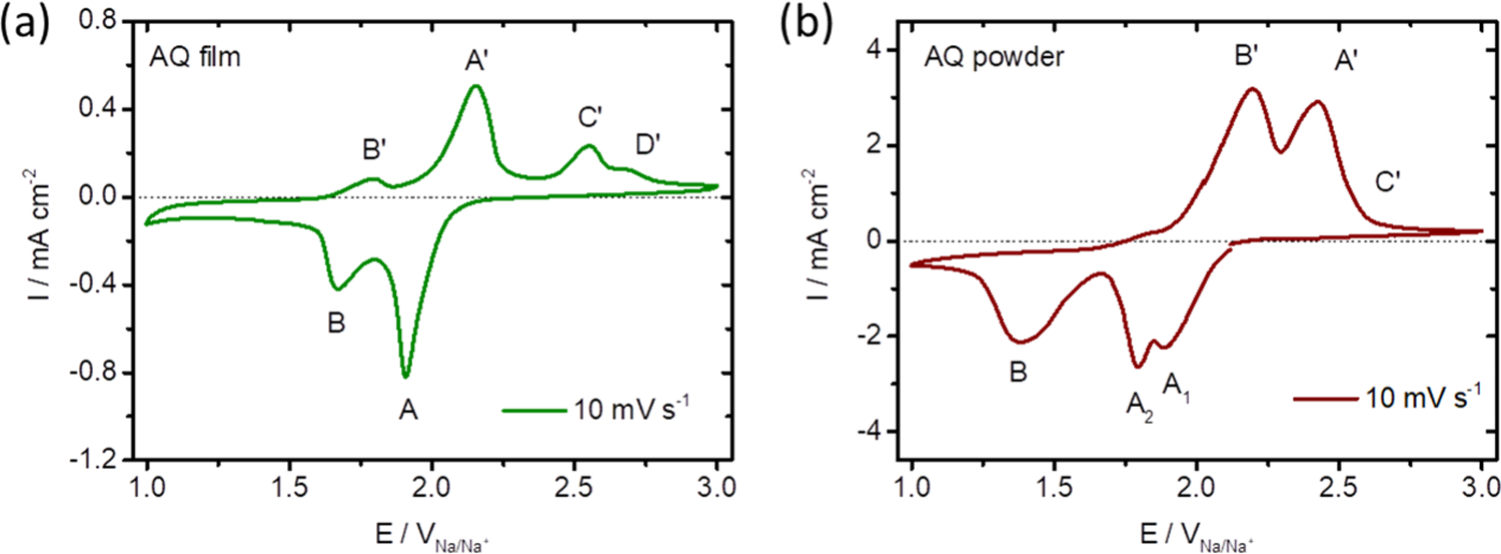
Cyclic voltammetry (CV) measurements of AQ thin-film (a, green line) and AQ powder (b, red line) electrodes at a scan rate of 10 mV s^–1^.

The apparent difference in morphology of the two different electrode structures is further reflected in the direct comparison of their electrochemical behavior. Cyclic voltammetry (CV) measurements are used to explore the electrochemical redox properties of both electrodes. Figure 3 depicts CV measurements of AQ thin-film and AQ powder electrodes in the potential range between 1.0 and 3.0 V, at a scan rate of 10 mV s^–1^.

Both CV measurements are characterized by broad reduction and oxidation waves. The AQ thin-film electrode (Figure 3a) shows two main peak pairs: one (Figure 3a, A/A′) with current maxima at 1.91/2.15 V (vs Na/Na^+^) and a second, less pronounced, at 1.67/1.80 V (B/B′). While the first peak pair (A/A′) can be attributed to the redox reaction of Na ions with the first carbonyl group of AQ, the second peak pair (B/B′) is most likely a combination of the reduction reaction of the second carbonyl group and the reduction of formed AQ dimers after the first reduction reaction.^17^ This is corroborated by the appearance of two additional oxidation waves at 2.55 V (C′) and 2.69 V (D′), next to the two back-oxidation peaks B′ and A′, which have been previously attributed to the oxidation of formed dimers following the first electron reduction reaction to the AQ radical anions.^17,36^ Their oxidation potential is well-shifted toward higher voltages since these dimers are stabilized by cations, such as H^+^ or Na^+^, present in the battery electrolyte solution.^36,37^ Interestingly, for the AQ powder electrode, the first reduction peak is characterized by two peak maxima (Figure 3b, peak A_1_ and A_2_), at 1.79 V (A_1_) and 1.88 V (A_2_), next to peak B with its peak current maxima at 1.38 V. Again, a dimerization reaction may explain the double-peak feature of the first reduction peak for the AQ powder electrode. This double-peak feature (A_1_ and A_2_) for the AQ powder electrode is only resolved at fast scan rates (>2 mV s^–1^, Figure S4), while, at the same time, the corresponding back-oxidation peak C becomes more pronounced at slower scan rates.

The peak separation (Δ*E*_P_) between the reduction and oxidation peak maxima is significantly smaller for the AQ thin-film electrodes compared to the powder-based AQ electrodes. A Δ*E*_P A–A′_ of 0.24 V and Δ*E*_P B–B′_ of 0.13 V is measured for the AQ thin-film electrodes, compared to a Δ*E*_P A–A′_ of 0.63 V and Δ*E*_P B–B′_ of 0.82 V for the AQ powder electrodes. In the thin-film configuration, the stacked AQ molecules, like pigment molecules in general, are expected to adopt tight π–π stacking along one crystallographic direction of the carrier substrate.^9,62^ This aligned orientation of the AQ molecules in the film is beneficial to obtain a good charge transport in the electrode film. The electric field, induced by the respective polarized electrodes, allows for the delocalization of charges through the already established π–π network,^1^ resulting in faster charge-transfer kinetics compared to the inordinate AQ molecules in the powder-based AQ electrodes. This stacking inducing faster charge-transfer kinetics in the AQ thin-film electrodes, consequently, results in a smaller Δ*E*_P_.^63^ The fact that no double-peak feature of the first reduction peak is observed for the AQ thin-film electrodes, different from the AQ powder electrodes (Figure 3), while at the same time two additional oxidation peaks C′ and D′ are present (Figure 3a), further corroborates this theory. Once formed, the dimers are stabilized by cations (Na^+^) present in the electrolyte and need, consequently, higher overpotentials to be back-oxidized again. They therefore only become visible (i.e., well-separated) in their oxidation peaks.

The shift toward a higher oxidation potential is characteristic of a barrier-induced charge transfer overpotential present in the thick (10 μm) powder-based matrix. This is also reflected in electrochemical impedance spectroscopy (EIS) performed on both systems. The impedance for the AQ thin-film and AQ powder electrodes at open circuit potential (OCP) is characterized by a well-defined semicircle at high frequencies in the corresponding Nyquist plots (Figure S5). This impedance is attributed to the charge transfer resistance (*R*_ct_), which is in the order of 88 Ω for AQ thin-film and 157 Ω for AQ powder electrodes. Although the *R*_ct_ for the AQ thin-film electrode is only half of that of the AQ powder electrode, if the *R*_ct_ is normalized to the overall film thickness, being 200 nm for the AQ thin film and 10 μm for the AQ powder, the specific *R*_ct_ is found to be about 28 times higher for the AQ thin film (44 × 10^7^–1.6 × 10^7^ Ω m^–1^). This is expected since in the AQ thin-film electrode no conductive carbon additive is used and AQ thin films are known to form a wide-band-gap semiconductor, having a band gap >3 eV.^58^ This is also corroborated by the absolute peak currents being about 10 times higher for the AQ powder electrodes (Figure 3).

In general, our CV measurements are in agreement with previous literature reports on AQ-based electrodes, in terms of redox potential, voltammogram shape, and electrochemical reversibility.^17,64,65^ Furthermore, the electrochemical investigations clearly demonstrate that the structural characteristics (i.e., stacking formation) and the chemical environment (i.e., binder and conductive carbon additives) of the AQ molecules have a substantial influence on their electrode performance. To deduce what might be the fundamental reason for this difference between thin-film and powder-based electrodes, our experimental studies were further augmented via computational investigations of different charged states of AQ on a carbon carrier substrate obtained via basin hopping global minimization using self-consistent charge density functional tight-binding (SCC DFTB) formalism.

### 3.3 Theoretical Calculations

The main driving forces of the crystal formation process in pigment molecules are either the intra- and intermolecular hydrogen bonds or the π–π stacking interactions between adjacent planar molecules, together with weak van der Waals interactions between chains of molecules.^1^ The overall effect of the aforementioned π–π stacking interactions generally leads to stabilization of the crystal lattice by lowering the energy of the stacked layers in the AQ crystal and the carbon carrier substrate. Especially, the interaction energy of the AQ molecules with the carbon carrier substrate (i.e., graphite) is an important parameter for the long-term stability of organic-based electrode materials and has important implications for the physicochemical properties of these highly complex systems at the molecular level. In the following sections, the calculation results obtained for AQ, AQ-Na, and AQ-Na_2_, as well as the respective dimers on a graphite model system, obtained via basin hopping global minimization at SCC DFTB/3ob level of theory, are presented. These systems are chosen since they represent the initial step in the film formation of the active AQ material. The obtained data provide detailed insight into the structural and associated energetic properties of the AQ-surface, as well as the intermolecular AQ–AQ contributions. To assess the suitability of the chosen DFTB level of theory, benchmark calculations of AQ on a single graphene layer against DFT calculations have been carried out and are included in the Supporting Information, Section S1.

#### 3.3.1 Monomers

**Figure 4:**
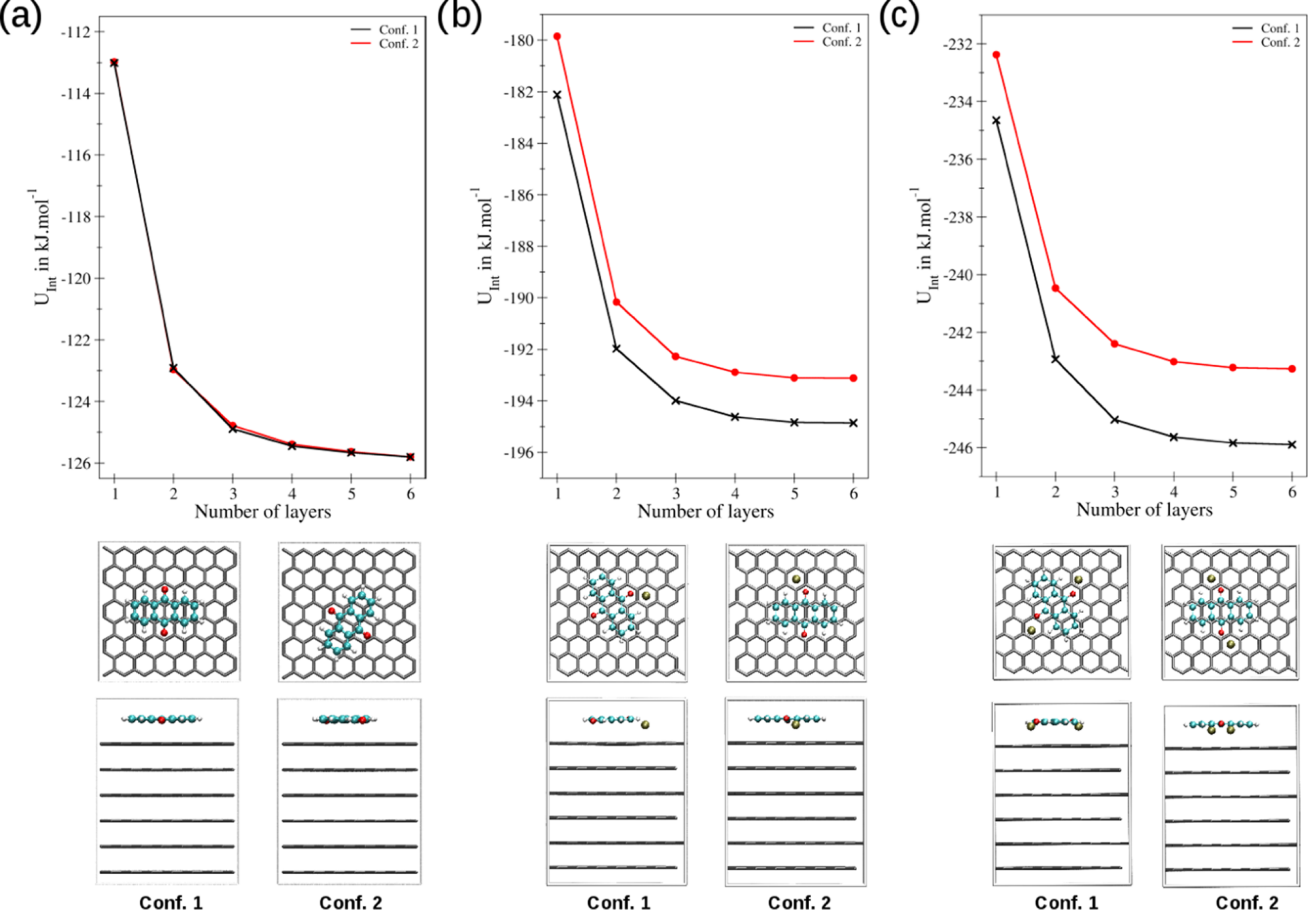
Convergence of the interaction energy of two best conformers identified for (a) AQ, (b) AQ-Na, and (c) AQ-Na_2_ as a function of the number of layers representing the carbon carrier substrate obtained at the SCC DFTB/3ob level of theory.

Figure 4a–c displays the dependence of the interaction energy *U*_int_ for the two best conformations identified via basin hopping for each of the three different molecular configurations (AQ, AQ-Na, and AQ-Na_2_) considered. In all three cases, a significant decrease in *U*_int_ is observed upon the increase in the number of layers representing the carbon carrier substrate, with the change from mono- to bilayer graphene having the strongest impact on the binding energy. Interestingly, a similar overall decrease in energy of approx. 13, 16, and 15 kJ mol^–1^ for the three different molecular configurations is observed when moving from a one- to six-layer description. For increments beyond *n* = 4, the interaction energies remain within 1 kJ mol^–1^ for all cases, and, hence, a four-layer system can be considered as an adequate compromise between the accuracy of results and computational effort.

In the case of the fully oxidized form of AQ, the best binding motifs display an ideally stacked conformation shifted by *a*_graph_/2 (see the experimental section) within the direction of the hexagonal lattice. Due to the overall apolar nature of the AQ molecule, the conformation parallel to the *a*-axis is effectively equal in energy as its rotated counterpart (i.e., Conf. 1 and 2 in Figure 4a). Along with the interaction energy, the average atom–layer distances are well-converged if at least four layers of the carrier material are included in the model system. While the carbon and hydrogen atoms of the AQ display very similar average atom–surface distances of 0.306 and 0.307 nm, the oxygen atoms are consistently found at smaller distances with the respective average value being 0.292 nm. This implies that the C=O bonds are slightly tilted toward the surface.

At first sight, a different situation is obtained when considering the reduced forms AQ-Na and AQ-Na_2_. The conformers rotated by 60° with respect to the *a*-axis appear lower in energy compared to the respective motifs aligned parallel to the *a* direction. However, this is a result of the periodic setup of the calculation system in conjunction with the long-range nature of Coulombic interactions. In the lower-energy motifs (Conf. 1) the ion–ion distance within the periodic cell is larger compared to the arrangement parallel to *a* (Conf. 2), which is an artifact introduced by the limited size of the calculation cell. However, aside from the differences in energy imposed by the cell geometry, the structural properties of these conformers are highly similar: in all cases, the ideally stacked arrangement of the fully oxidized AQ molecule is retained. Interestingly, the Na^+^–O distance enables the ions to position close to the center of a six-membered ring structure at the surface of the carrier, which results in an average surface–Na^+^ distance of approx. 0.235 nm. This, on the other hand, has a minor impact on the average molecule–surface distance. While the increase in the average surface–C and surface–H distances is in the range of at most 0.003 nm, the average surface–O distance is increased by 0.08 and 0.023 nm upon one- and two-fold reductions of AQ. This implies that in the fully reduced form, the C–O^–^ bonds display a slight tilt away from the surface.

The magnitude of the calculated molecular–surface interaction energy *U*_int_ is effectively doubled when moving from the oxidized to the fully reduced form of the AQ moiety, which results from the increased Coulombic character of the interaction. This finding appears to be counterintuitive and contradictory to the results of the experimental measurements, showing that the reduced species show a higher desorption rate. However, the adsorption at the surface is in competition with the associated solvation process and, typically, the free energy of solvation Δ*G*_solv_ is dramatically increased in magnitude (i.e., showing more negative values) if the species in question carries a net charge (e.g., compare the hydration free energy of simple monovalent ions^66,67^ with that of an apolar molecule,^68^ such as CO_2_). This is typically true in the case of the associated enthalpy of solvation Δ*H*_solv_ and in many cases also in terms of the entropic perspective Δ*S*_solv_.^69^ Especially, when considering polar solvents, the dissolution of Na ions and the reduced AQ moiety can be expected to be favorable in both the enthalpic and the entropic contributions.

As a consequence, the data presented in this study can only provide information with respect to the mode of binding and the associated structural properties of the active molecules at the surface of the carbon carrier substrate. However, without consideration of the species in solution, no information about the actual equilibrium between adsorption and solution can be given, since the latter depends strongly on the solubility of the species in the given solvent, which may be further influenced by local thermal effects.

#### 3.3.2 Dimers

**Figure 5:**
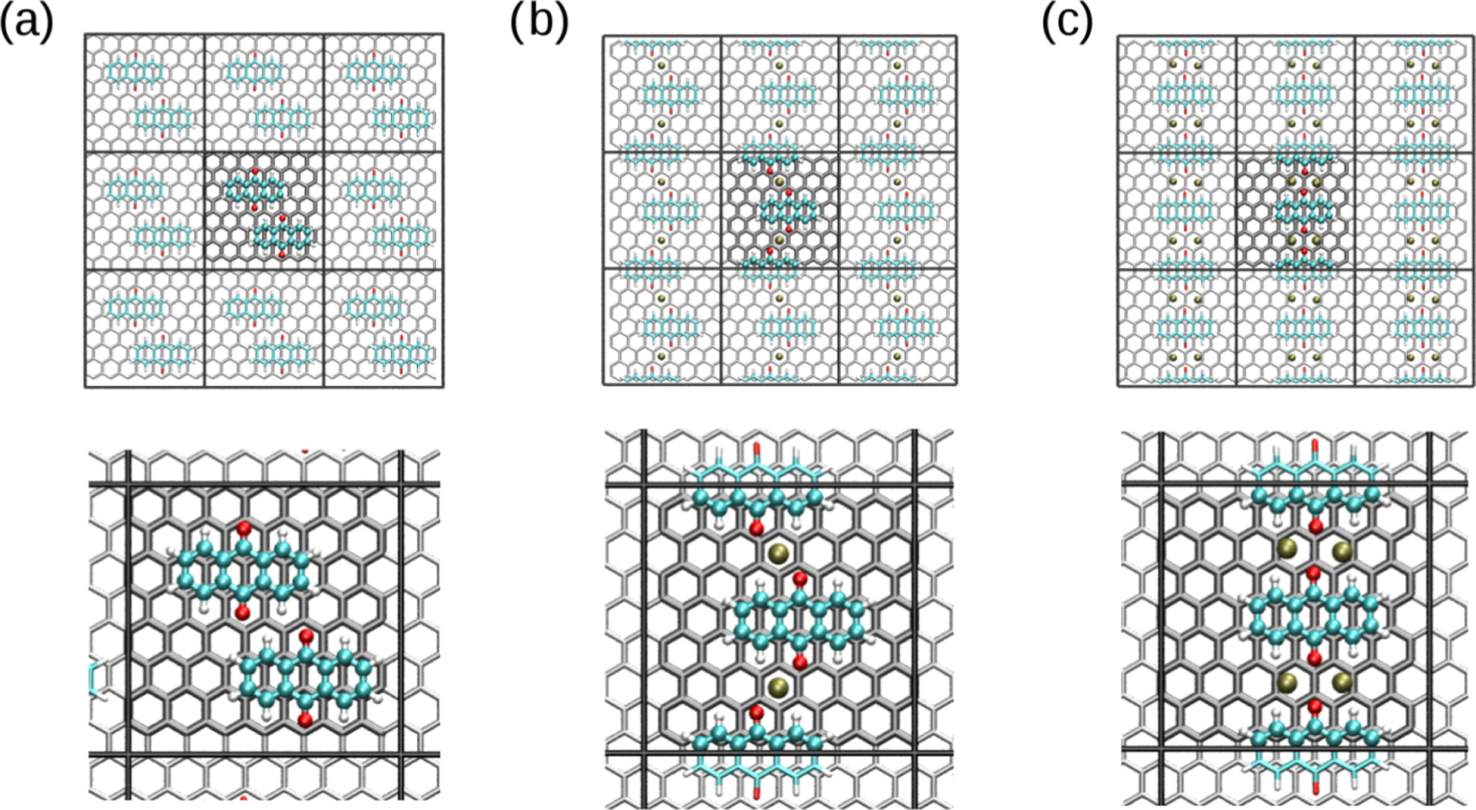
Best surface motifs identified for the dimers of (a) AQ, (b) AQ-Na, and (c) AQ-Na_2_ on a four-layer representation of the carbon carrier obtained at the SCC DFTB/3ob level of theory.

Upon electrochemical investigation, intermolecular dimerization of AQ molecules can be inferred, suggesting that the charged state of the formed dimers is dependent on the chemical surroundings. To determine how the formation of dimers may impact the charge-storage characteristics of the AQ thin-film and powder-based electrodes, theoretical calculations examining the ordering effects of the respective dimer systems in a periodic supercell environment are performed. Figure 5a–c depicts the ideal conformers identified for the dimer chains of AQ, AQ-Na, and AQ-Na_2_, respectively. In each case, a four-layer representation of the carrier is considered, since it provides an adequate compromise between the accuracy of results and computational demand.

In the case of the fully oxidized form, the best structural arrangement at the surface still displays the stacked conformation observed for the individual monomers, while, at the same time, two intermolecular hydrogen bonds between the oxygen atom of one molecule and the C–H group of the second AQ can be identified. As a consequence, the two molecular units are located at an offset of two unit cells (i.e., 2*a*_graph_) along the *a* direction. Although the H-bonds do not display an ideal linear arrangement and a C–H group has to be considered as a very weak H-bond donor, this particular conformation displays the lowest interaction energy of −270.9 kJ mol^–1^ when compared to other possible conformers of the molecules at the surface (data not shown).

It should be noted that the interaction energy, *U*_int_, obtained according to eq 1, considers not only the molecule–surface interactions but also the respective AQ–AQ contributions. Comparing this value with two times *U*_int_ obtained for the best monomeric structure on a four-layer carrier given as −250.8 kJ mol^–1^ results in a difference of −20.1 kJ mol^–1^. The latter value agrees well with the interaction energy between two energy-minimized AQ molecules of −20.8 kJ mol^–1^ (in the same periodic environment but in the absence of the carrier substrate), implying that the binding to the surface results in a minor rearrangement of the dimer linked to slightly unfavorable molecular interactions.

The ideal conformer of the dimer in the AQ-Na case shows some similarities to the fully reduced form discussed above. In this case, the two molecular units are shifted by only one unit cell (i.e., *a*_graph_) in the *a* direction, while being moved apart by one unit along the *b*′ direction to accommodate the two Na ions (see Figure 5b). The latter again occupy the central position of a six-membered ring of the carrier, as seen earlier for the respective monomer. It can be seen that the combined dimeric units form a 1D chain of molecules along the surface, which explains this particular choice of the cell dimensions for the system representing the carrier material, i.e., each AQ-Na unit occupies an equivalent of 4 unit cells along the *b*′ direction.

The associated total interaction energy of −608.4 kJ mol^–1^ is considerably more negative than two times the value of the respective AQ-Na monomer in the associated orientation (i.e., Conf. 2 in Figure 4b) of −385.9 kJ mol^–1^, indicating a strong cooperative effect in this particular configuration. The respective difference of −222.5 kJ mol^–1^ can again be attributed to the molecular AQ–AQ interaction, which, due to the associated ion–dipole interaction, is considerably stronger compared to the fully oxidized form. However, the latter value is significantly higher than the corresponding AQ–AQ interaction energy of −271.4 kJ mol^–1^ obtained for the same system minimized in the absence of the carrier. The associated difference of nearly 50 kJ mol^–1^ is due to the rearrangement of the AQ molecules at the surface of the carbon substrate.

Finally, the dimer of the fully reduced form is considered, in which case a notable difference in the structure is observed. Each AQ-Na_2_ unit now occupies 5 unit cells along the *b*′ direction. However, the respective regions for the individual moieties intersect, and the entire dimer structure can be accommodated within 8 unit cells along the *b*′ direction. Inspection of the optimized structure reveals that the AQ residues are not shifted along the *a* direction anymore but arranged in a parallel fashion (see Figure 5c). This is in contrast to the ideal structures for the oxidized and partially reduced forms discussed above. The reason for this new structural motif results from the fact that the Na ions are now arranged on both sides of the C–O^–^ groups, thus forming two salt bridges between the oxygen atoms of the two AQ residues. The Na ions are also not located at the ideal positions in the center of a six-membered ring at the surface, which is a result of their close vicinity and the associated Coulombic repulsion. Of all of the dimer structures considered, this motif showed the most negative interaction energy of −717.8 kJ mol^–1^, which is again lower than two times the value of the associated monomer, being −486.0 kJ mol^–1^. In this case, the difference amounts only to −231.8 kJ mol^–1^ as opposed to interaction energy of −360.1 kJ mol^–1^ obtained for AQ_2_-Na_4_ in the absence of the carrier, which implies a significant loss in the molecular interaction energy of 128.3 kJ mol^–1^ upon binding to the surface. This loss in the molecular interaction energy could explain why, in the thin-film configuration, the dimers remain electronically active over long-term galvanostatic cycling, while the dimers that are formed in the powder-based system are too stabilized to be electronically accessible. In general, the calculated results obtained in this study clearly demonstrate the benefit of augmenting experimental studies via computational investigations and form the basis for further theoretical studies, thereby considering enlarged unit cells to accommodate a larger number of active molecules, as well as the formation of stacked configurations of these molecules on the carrier substrate material.

In summary, our theoretical calculations suggest the transition from intermolecular hydrogen bonding toward the formation of salt bridges between the reduced AQ units accompanied by a stabilizing effect upon a dimerlike rearrangement. In addition, the strong surface–molecular interactions in the thin-film geometries are found to be crucial for the formed dimers to remain electronically active. This dimerization, in combination with long-range order due to extensive intermolecular π–π interactions, results in a decent charge carrier transport and current collector adhesion, both of which are expected to be of mutual benefit for potential applications in secondary ion batteries.

### 3.4 Sodium Ion Battery Half-Cell Cycling

**Figure 6:**
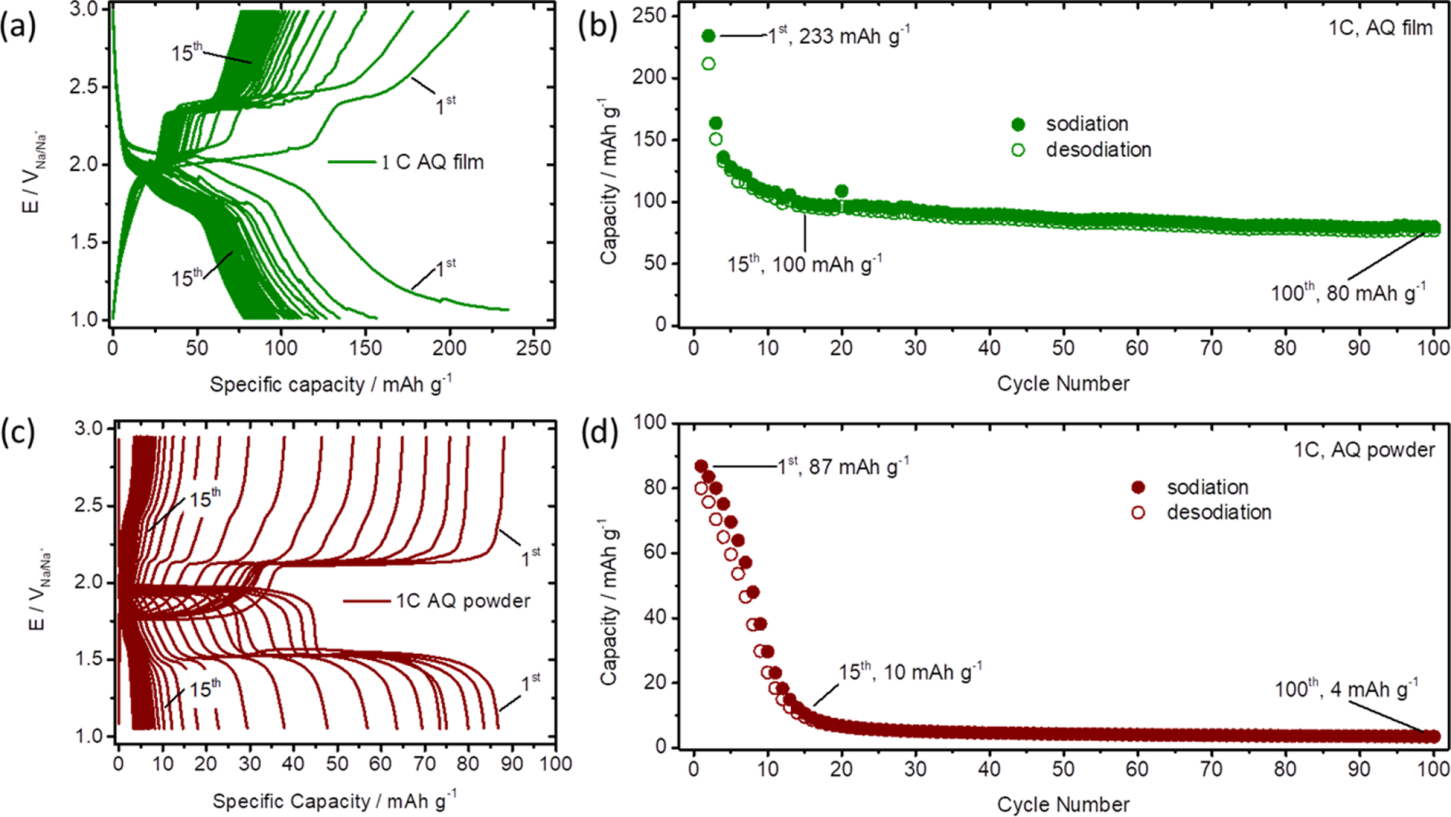
Comparison of the long-term galvanostatic constant current cycling measurements and the corresponding specific capacity (charge, open circles and discharge, closed circles) over 100 sodiation/desodiation cycles for (a, b) AQ thin-film and (c, d) AQ powder electrodes at a 1C rate from 3.0 to 1.0 V.

To investigate the potential application of the ordering in AQ stacking, forced by thin-film application, and its effect on dimer solubility and current collector adhesion, Na ion battery cycling was performed. Long-term galvanostatic constant current battery half-cell cycling (GCPL) of AQ thin-film and AQ powder electrodes at a 1C rate, with C being defined as the maximum theoretical capacity (being 257 mAh g^–1^) obtained in 1 h, is shown in Figure 6.

Figure 6a,c compares the galvanostatic cycling characteristics for AQ thin-film (Figure 6a) and AQ powder electrodes (Figure 6c). The discharging behavior (cell voltage upon sodiation) for the AQ thin-film electrode is characterized by a sloping curve (i.e., a varying cell voltage), while the AQ powder electrode shows distinct voltage plateaus (i.e., a stable cell voltage) at about 2.0 and 1.5 V upon sodiation and 1.8 and 2.1 V upon desodiation. This difference in the shape of both the CV response as well as the discharging cell voltage, between the AQ thin-film and AQ powder electrodes, can be explained by the morphology difference of the two electrodes. In the thin-film configuration, the stacked AQ molecules interact via their π-system. Upon electrochemical reduction or oxidation, the π-system is capable of storing electric energy as delocalized charge carriers, similar to conjugated polymers.^70,71^ Therefore, the AQ thin-film electrode has no sharp redox potential (different from conventional battery electrode materials). Instead, the AQ thin-film electrode demonstrates a floating potential that is altered by the degree of sodiation. As the degree of sodiation changes upon charging/discharging, a constant change of the half-cell voltage is implied (Figure 6a), resulting in the observed sloping voltage curve. Since the charged AQ molecules are electronically more separated in the powder-based electrodes, only small domains of intermolecular π–π stacked AQ are present; hence, the sodiation level does not significantly affect the molecules. Due to the isolated redox-active AQ unit, the powder-based electrodes feature redox reactions at a very distinct potential and, consequently, demonstrate voltage profiles upon sodiation with stable charge/discharge voltages (Figure 6c). A similar effect has been previously reported for redox polymers having a nonconducting backbone in combination with distinct redox units attached to them.^72−74^ Different from state-of-the-art conducting polymers,^71,75^ the redox polymers are characterized by a stable voltage plateau upon charge/discharge cycling.

The AQ thin-film electrode exhibits a specific sodiation capacity of 233 mAh g^–1^ for the first and 163 mAh g^–1^ for the second sodiation/desodiation cycle. This is substantially higher compared to the initial specific sodiation capacity of the AQ powder electrode, being 87 mAh g^–1^ for the first and 83 mAh g^–1^ for the second sodiation/desodiation cycle. Additionally, in terms of stability, 43% of the initial AQ thin-film electrode capacity (100 mAh g^–1^) is found to be still accessible after 15 and 34% (80 mAh g^–1^) after 100 and 26% (67 mAh g^–1^, Figure S6), after 250 sodiation/desodiation cycles at a rate of 1C. The decrease in capacity measured in the galvanostatic cycling experiments can be attributed to three main deactivation mechanisms: chemical degradation (i.e., dimerization), swelling of the electrode, and dissolution of the active species.^76^ It is interesting to notice that the long-term stable specific capacity of the AQ thin-film electrode, being about one-third of the initial capacity, would fit the specific capacity of a transition from AQ monomers (2e^–^ per AQ with *C*_th_ of 257 mAh g^–1^) to AQ dimers (2e^–^ per 2AQ with *C*_th_ of 128 mAh g^–1^), with some additional, minor material loss upon swelling of the electrode and dissolution. Dimerization is a known possibility to suppress the solubility of organic molecules, which has been utilized before. For example, in a recent study, Yao et al. were able to show that AQ material dissolution can be substantially mitigated if simple AQ is replaced by the synthesized dimer (AQ-C White Heart Suit C-AQ).^37^ Their reported capacity retention for the dimer, of approx. 31% after 100 cycles, is remarkably similar to our results of 34% over the same cycle numbers for the AQ thin-film electrodes.

Differently, for the AQ powder electrode, only 11% of the initial capacity (10 mAh g^–1^) is still accessible after 15 and almost none (5% or 4 mAh g^–1^) after 100 sodiation/desodiation cycles. Not surprisingly, the AQ thin-film electrode in this work exhibits higher Coulombic efficiencies (CEs) compared to the AQ powder electrode in the course of 100 sodiation/desodiation cycles (Figure S7). While the CE for the AQ thin-film electrode is 97.2% in the 15th sodiation/desodiation cycle, it drops to a minimum of only 82.9% for the AQ powder electrode. Consequently, the GCPL measurements show higher specific gravimetric Na storage capacities and significantly better long-term stabilities of the AQ thin-film electrodes over AQ powder electrodes. Therefore, SIB half-cell cycling results in this work confirm the conclusions drawn from the theoretical calculations and provide encouraging results that invite future exploration of thin-film AQ electrodes for their potential application in various metal ion batteries.

## 4 Conclusions

In conclusion, we found that the structural characteristics (i.e., crystal formation) and the chemical environment (i.e., binder and conductive carbon additives) of the AQ molecules have a substantial influence on their electrochemical performance. IR analysis reveals significantly stronger π–π interactions and a lower degree of disorder in the AQ thin-film over the AQ powder electrodes. This has been further augmented by computational investigations of different charged states of AQ on a carbon carrier substrate obtained via basin hopping global minimization at the SCC DFTB/3ob level. Examining the ordering effects of the different molecular species employing the respective dimerlike systems in a periodic supercell environment enabled the decomposition of the associated energetic contributions into surface–molecular and molecular–molecular interactions. Both are found to increase in magnitude upon reduction of the AQ moiety, thereby reflecting the transition from intermolecular hydrogen bonding toward the formation of salt bridges between the reduced AQ units. Of all of the different structures considered, the dimerlike structure of the fully reduced form (AQ_2_-Na_4_) is found to show the most negative interaction energy with the carbon substrate in combination with a significant loss in the molecular interaction energy upon binding to the surface. This weakening of the intermolecular interaction energy is essential for the formed dimer chains to remain electronically active toward Na ion storage. The stronger π–π interactions, in combination with differences in morphology of the AQ thin-film over the AQ powder electrodes, are further reflected in the direct comparison of their SIB performance. It is found that the AQ thin-film electrode exhibits significantly better specific capacities (233 vs 87 mAh g^–1^ in the first cycle), Coulombic efficiencies, and long-term cycling performance (80 vs 4 mAh g^–1^ after 100 cycles) over the AQ powder electrode. Additionally, both CV and galvanostatic cycling point toward the formation of AQ dimers upon repeated battery cycling in the thin-film electrode, corroborating the theoretical calculations. The additional stabilization upon dimerization may explain the better cycling stability of the AQ thin-film over the AQ powder electrode. Therefore, future studies on powder-based electrode materials should focus on combining the high conductivity in the powder matrix with the strong surface–molecular interactions in thin-film geometries. In combination, this may foster the performance of industrially desirable powder-based electrode materials for secondary batteries.

## Supporting Information Available

Unit cell of the surface model and the sketch of the region employed to generate starting configurations for the conformation search via basin hopping (Figure S1); scanning electron micrographs (SEM) of AQ thin-film and AQ powder electrodes (Figure S2); ATR-FTIR spectra of AQ thin film, AQ powder, and pure carbon carrier substrate electrodes (Figure S3); cyclic voltammograms of AQ powder electrodes at scan rates of 2 and 0.5 mV s^–1^ (Figure S4); Nyquist plots of AQ thin-film (green line) and AQ powder (red line) electrodes (Figure S5); long-term specific capacity and the corresponding calculated efficiency vs cycle number over 250 sodiation/desodiation cycles for AQ thin-film electrodes (Figure S6); comparison of the long-term specific capacity and the corresponding calculated efficiency vs cycle number (Figure S7); detailed description of DFTB vs DFT (Section S1); and interaction energy and average atom–surface distance for C, H, and O atoms obtained for AQ on graphene at different levels of theory (Table S1) (PDF)

## References

[ref1] Irimia-Vladu M.; Kanbur Y.; Camaioni F.; Coppola M. E.; Yumusak C.; Irimia C. V.; Vlad A.; Operamolla A.; Farinola G. M.; Suranna G. P.; González-Benitez N.; et al. Stability of Selected Hydrogen Bonded Semiconductors in Organic Electronic Devices. Chem. Mater. 2019, 31, 6315–6346. 10.1021/acs.chemmater.9b01405. 32565617 PMC7297463

[ref2] Głowacki E. D.; Voss G.; Leonat L.; Irimia-Vladu M.; Bauer S.; Sariciftci N. S. Indigo and Tyrian Purple - From Ancient Natural Dyes to Modern Organic Semiconductors. Isr. J. Chem. 2012, 540–551. 10.1002/ijch.201100130.

[ref3] Głowacki E. D.; Irimia-Vladu M.; Bauer S.; Sariciftci N. S. Hydrogen-Bonds in Molecular Solids – from Biological Systems to Organic Electronics. J. Mater. Chem. B 2013, 1, 3742 10.1039/c3tb20193g. 32261127

[ref4] Irimia-Vladu M.; Głowacki E. D.; Troshin P. A.; Schwabegger G.; Leonat L.; Susarova D. K.; Krystal O.; Ullah M.; Kanbur Y.; Bodea M. A.; et al. Indigo - A Natural Pigment for High Performance Ambipolar Organic Field Effect Transistors and Circuits. Adv. Mater. 2012, 24, 375–380. 10.1002/adma.201102619. 22109816

[ref5] Miroshnikov M.; Divya K. P.; Babu G.; Meiyazhagan A.; Reddy Arava L. M.; Ajayan P. M.; John G. Power from Nature: Designing Green Battery Materials from Electroactive Quinone Derivatives and Organic Polymers. J. Mater. Chem. A 2016, 4, 12370–12386. 10.1039/C6TA03166H.

[ref6] Armand M.; Grugeon S.; Vezin H.; Laruelle S.; Ribière P.; Poizot P.; Tarascon J.-M. Conjugated Dicarboxylate Anodes for Li-Ion Batteries. Nat. Mater. 2009, 8, 120–125. 10.1038/nmat2372. 19151701

[ref7] Lincke G. Molecular Stacks as a Common Characteristic in the Crystal Lattice of Organic Pigment Dyes A Contribution to the “Soluble–Insoluble” Dichotomy of Dyes and Pigments from the Technological Point of View. Dyes Pigm. 2003, 59, 1–24. 10.1016/S0143-7208(03)00097-4.

[ref8] Winkler C.; Mayer F.; Zojer E. Analyzing the Electronic Coupling in Molecular Crystals—The Instructive Case of A-Quinacridone. Adv. Theory Simul. 2019, 2, 1800204 10.1002/adts.201800204.

[ref9] Głowacki E. D.; Irimia-Vladu M.; Kaltenbrunner M.; Gsiorowski J.; White M. S.; Monkowius U.; Romanazzi G.; Suranna G. P.; Mastrorilli P.; Sekitani T.; et al. Hydrogen-Bonded Semiconducting Pigments for Air-Stable Field-Effect Transistors. Adv. Mater. 2013, 25, 1563–1569. 10.1002/adma.201204039. 23239229

[ref10] Liebl S.; Werner D.; Apaydin D. H.; Wielend D.; Geistlinger K.; Portenkirchner E. Perylenetetracarboxylic Diimide as Diffusion-Less Electrode Material for High-Rate Organic Na-Ion Batteries. Chem. - Eur. J. 2020, 26, 17559–17566. 10.1002/chem.202003624. 32767398 PMC7839514

[ref11] Liang Y.; Yao Y. Positioning Organic Electrode Materials in the Battery Landscape. Joule 2018, 2, 1690–1706. 10.1016/j.joule.2018.07.008.

[ref12] Lu Y.; Chen J. Prospects of Organic Electrode Materials for Practical Lithium Batteries. Nat. Rev. Chem. 2020, 4, 127–142. 10.1038/s41570-020-0160-9. 37128020

[ref13] Han C.; Li H.; Shi R.; Zhang T.; Tong J.; Li J.; Li B. Organic Quinones towards Advanced Electrochemical Energy Storage: Recent Advances and Challenges. J. Mater. Chem. A 2019, 7, 23378–23415. 10.1039/C9TA05252F.

[ref14] Larcher D.; Tarascon J.-M. Towards Greener and More Sustainable Batteries for Electrical Energy Storage. Nat. Chem. 2015, 7, 19–29. 10.1038/nchem.2085. 25515886

[ref15] Dražević E.; Andersen A. S.; Wedege K.; Henriksen M. L.; Hinge M.; Bentien A. Investigation of Low-Cost Oligoanthraquinones for Alkaline, Aqueous Rechargeable Batteries with Cell Potential up to 1.13 V. J. Power Sources 2018, 381, 94–100. 10.1016/j.jpowsour.2018.01.092.

[ref16] Bitenc J.; Pavčnik T.; Košir U.; Pirnat K. Quinone Based Materials as Renewable High Energy Density Cathode Materials for Rechargeable Magnesium Batteries. Materials 2020, 13, 506 10.3390/ma13030506. PMC7040669 31973193

[ref17] Werner D.; Apaydin D. H.; Portenkirchner E. An Anthraquinone/Carbon Fiber Composite as Cathode Material for Rechargeable Sodium-Ion Batteries. Batteries Supercaps 2018, 1, 160–168. 10.1002/batt.201800057.

[ref18] Zhang Y.; Murtaza I.; Liu D.; Tan R.; Zhu Y.; Meng H. Understanding the Mechanism of Improvement in Practical Specific Capacity Using Halogen Substituted Anthraquinones as Cathode Materials in Lithium Batteries. Electrochim. Acta 2017, 224, 622–627. 10.1016/j.electacta.2016.12.065.

[ref19] Yao M.; Yamazaki S. I.; Senoh H.; Sakai T.; Kiyobayashi T. Crystalline Polycyclic Quinone Derivatives as Organic Positive-Electrode Materials for Use in Rechargeable Lithium Batteries. Mater. Sci. Eng., B 2012, 177, 483–487. 10.1016/j.mseb.2012.02.007.

[ref20] Li W.; Chen L.; Sun Y.; Wang C.; Wang Y.; Xia Y. All-Solid-State Secondary Lithium Battery Using Solid Polymer Electrolyte and Anthraquinone Cathode. Solid State Ionics 2017, 300, 114–119. 10.1016/j.ssi.2016.12.013.

[ref21] Wang W.; Xu W.; Cosimbescu L.; Choi D.; Li L.; Yang Z. Anthraquinone with Tailored Structure for a Nonaqueous Metal–Organic Redox Flow Battery. Chem. Commun. 2012, 48, 6669 10.1039/c2cc32466k. 22641051

[ref22] Xu F.; Xia J.; Shi W.; Cao S. Electrochemical Properties of Anthraquinone-Based Polyimides as Cathodes for Lithium Secondary Batteries. Chem. Lett. 2016, 45, 271–273. 10.1246/cl.151020.

[ref23] Kawai T.; Oyaizu K.; Nishide H. High-Density and Robust Charge Storage with Poly(Anthraquinone-Substituted Norbornene) for Organic Electrode-Active Materials in Polymer–Air Secondary Batteries. Macromolecules 2015, 48, 2429–2434. 10.1021/ma502396r.

[ref24] Zhao J.; Yang J.; Sun P.; Xu Y. Sodium Sulfonate Groups Substituted Anthraquinone as an Organic Cathode for Potassium Batteries. Electrochem. Commun. 2018, 86, 34–37. 10.1016/j.elecom.2017.11.009.

[ref25] Hwang J.-Y.; Myung S.-T.; Sun Y.-K. Sodium-Ion Batteries: Present and Future. Chem. Soc. Rev. 2017, 46, 3529–3614. 10.1039/C6CS00776G. 28349134

[ref26] Slater M. D.; Kim D.; Lee E.; Johnson C. S. Sodium-Ion Batteries. Adv. Funct. Mater. 2013, 23, 947–958. 10.1002/adfm.201200691.

[ref27] Vaalma C.; Buchholz D.; Weil M.; Passerini S. A Cost and Resource Analysis of Sodium-Ion Batteries. Nat. Rev. Mater. 2018, 3, 18013 10.1038/natrevmats.2018.13.

[ref28] Holleman A.; Wiberg N. Lehrbuch Der Anorganischen Chemie, 102nd ed.; De Gruyter: Berlin, 2007.

[ref29] Nayak P. K.; Yang L.; Brehm W.; Adelhelm P. From Lithium-Ion to Sodium-Ion Batteries: Advantages, Challenges, and Surprises. Angew. Chem., Int. Ed. 2018, 57, 102–120. 10.1002/anie.201703772. 28627780

[ref30] Chayambuka K.; Mulder G.; Danilov D. L.; Notten P. H. L. Sodium-Ion Battery Materials and Electrochemical Properties Reviewed. Adv. Energy Mater. 2018, 8, 1800079 10.1002/aenm.201800079.

[ref31] Grey C. P.; Tarascon J. M. Sustainability and in Situ Monitoring in Battery Development. Nat. Mater. 2017, 16, 45–56. 10.1038/nmat4777. 27994251

[ref32] Phadke S.; Cao M.; Anouti M. Approaches to Electrolyte Solvent Selection for Poly-Anthraquinone Sulfide Organic Electrode Material. ChemSusChem 2018, 11, 965–974. 10.1002/cssc.201701962. 29205911

[ref33] Schon T. B.; McAllister B. T.; Li P.-F.; Seferos D. S. The Rise of Organic Electrode Materials for Energy Storage. Chem. Soc. Rev. 2016, 45, 6345–6404. 10.1039/C6CS00173D. 27273252

[ref34] Tang W.; Liang R.; Li D.; Yu Q.; Hu J.; Cao B.; Fan C. Highly Stable and High Rate-Performance Na-Ion Batteries Using Polyanionic Anthraquinone as the Organic Cathode. ChemSusChem 2019, 12, 2181–2185. 10.1002/cssc.201900539. 30896083

[ref35] Li D.; Tang W.; Yong C. Y.; Tan Z. H.; Wang C.; Fan C. Long-Lifespan Polyanionic Organic Cathodes for Highly Efficient Organic Sodium-Ion Batteries. ChemSusChem 2020, 13, 1991–1996. 10.1002/cssc.202000131. 32057185

[ref36] Carney T. J.; Collins S. J.; Moore J. S.; Brushett F. R. Concentration-Dependent Dimerization of Anthraquinone Disulfonic Acid and Its Impact on Charge Storage. Chem. Mater. 2017, 29, 4801–4810. 10.1021/acs.chemmater.7b00616.

[ref37] Yao M.; Sano H.; Ando H.; Kiyobayashi T.; Takeichi N. Anthraquinone-Based Oligomer as a Long Cycle-Life Organic Electrode Material for Use in Rechargeable Batteries. ChemPhysChem 2019, 20, 967–971. 10.1002/cphc.201900012. 30775839

[ref38] Poizot P.; Dolhem F.; Gaubicher J. Progress in All-Organic Rechargeable Batteries Using Cationic and Anionic Configurations: Toward Low-Cost and Greener Storage Solutions?. Curr. Opin. Electrochem. 2018, 9, 70–80. 10.1016/j.coelec.2018.04.003.

[ref39] Elstner M.; Porezag D.; Jungnickel G.; Elsner J.; Haugk M.; Frauenheim T.; Suhai S.; Seifert G. Self-Consistent-Charge Density-Functional Tight-Binding Method for Simulations of Complex Materials Properties. Phys. Rev. B 1998, 58, 7260–7268. 10.1103/PhysRevB.58.7260.

[ref40] Yang Y.; Yu H.; York D.; Cui Q.; Elstner M. Extension of the Self-Consistent-Charge Density-Functional Tight-Binding Method: Third-Order Expansion of the Density Functional Theory Total Energy and Introduction of a Modified Effective Coulomb Interaction. J. Phys. Chem. A 2007, 111, 10861–10873. 10.1021/jp074167r. 17914769

[ref41] Gaus M.; Goez A.; Elstner M. Parametrization and Benchmark of DFTB3 for Organic Molecules. J. Chem. Theory Comput. 2013, 9, 338–354. 10.1021/ct300849w. 26589037

[ref42] Kubillus M.; Kubař T.; Gaus M.; Řezáč J.; Elstner M. Parameterization of the DFTB3 Method for Br, Ca, Cl, F, I, K, and Na in Organic and Biological Systems. J. Chem. Theory Comput. 2015, 11, 332–342. 10.1021/ct5009137. 26889515

[ref43] Grimme S.; Antony J.; Ehrlich S.; Krieg H. A Consistent and Accurate Ab Initio Parametrization of Density Functional Dispersion Correction (DFT-D) for the 94 Elements H-Pu. J. Chem. Phys. 2010, 132, 154104 10.1063/1.3382344. 20423165

[ref44] Grimme S.; Ehrlich S.; Goerigk L. Effect of the Damping Function in Dispersion Corrected Density Functional Theory. J. Comput. Chem. 2011, 32, 1456–1465. 10.1002/jcc.21759. 21370243

[ref45] Hourahine B.; Aradi B.; Blum V.; Bonafé F.; Buccheri A.; Camacho C.; Cevallos C.; Deshaye M. Y.; Dumitrică T.; Dominguez A.; et al. DFTB+, a Software Package for Efficient Approximate Density Functional Theory Based Atomistic Simulations. J. Chem. Phys. 2020, 152, 124101 10.1063/1.5143190. 32241125

[ref46] Humphrey W.; Dalke A.; Schulten K. VMD: Visual Molecular Dynamics. J. Mol. Graphics 1996, 14, 33–38. 10.1016/0263-7855(96)00018-5. 8744570

[ref47] Yang G.; Li L.; Lee W. B.; Ng M. C. Structure of Graphene and Its Disorders: A Review. Sci. Technol. Adv. Mater. 2018, 19, 613–648. 10.1080/14686996.2018.1494493. 30181789 PMC6116708

[ref48] Wales D. J.; Doye J. P. K. Global Optimization by Basin-Hopping and the Lowest Energy Structures of Lennard-Jones Clusters Containing up to 110 Atoms. J. Phys. Chem. A 1997, 101, 5111–5116. 10.1021/jp970984n.

[ref49] Dovesi R.; Erba A.; Orlando R.; Zicovich-Wilson C. M.; Civalleri B.; Maschio L.; Rérat M.; Casassa S.; Baima J.; Salustro S.; Kirtman B. Quantum-Mechanical Condensed Matter Simulations with CRYSTAL. Wiley Interdiscip. Rev.: Comput. Mol. Sci. 2018, 8, e1360 10.1002/wcms.1360.

[ref50] Perdew J. P.; Ruzsinszky A.; Csonka G. I.; Vydrov O. A.; Scuseria G. E.; Constantin L. A.; Zhou X.; Burke K. Restoring the Density-Gradient Expansion for Exchange in Solids and Surfaces. Phys. Rev. Lett. 2008, 100, 136406 10.1103/PhysRevLett.100.136406. 18517979

[ref51] Weintraub E.; Henderson T. M.; Scuseria G. E. Long-Range-Corrected Hybrids Based on a New Model Exchange Hole. J. Chem. Theory Comput. 2009, 5, 754–762. 10.1021/ct800530u. 26609580

[ref52] Schimka L.; Harl J.; Kresse G. Improved Hybrid Functional for Solids: The HSEsol Functional. J. Chem. Phys. 2011, 134, 024116 10.1063/1.3524336. 21241089

[ref53] Becke A. D. Density-functional Thermochemistry. III. The Role of Exact Exchange. J. Chem. Phys. 1993, 98, 5648–5652. 10.1063/1.464913.

[ref54] Peintinger M. F.; Oliveira D. V.; Bredow T. Consistent Gaussian Basis Sets of Triple-Zeta Valence with Polarization Quality for Solid-State Calculations. J. Comput. Chem. 2013, 34, 451–459. 10.1002/jcc.23153. 23115105

[ref55] Vilela Oliveira D.; Laun J.; Peintinger M. F.; Bredow T. BSSE-correction Scheme for Consistent Gaussian Basis Sets of Double- and Triple-zeta Valence with Polarization Quality for Solid-state Calculations. J. Comput. Chem. 2019, 40, 2364–2376. 10.1002/jcc.26013. 31260123

[ref56] Vizintin A.; Bitenc J.; Kopač Lautar A.; Pirnat K.; Grdadolnik J.; Stare J.; Randon-Vitanova A.; Dominko R. Probing Electrochemical Reactions in Organic Cathode Materials via in Operando Infrared Spectroscopy. Nat. Commun. 2018, 9, 661 10.1038/s41467-018-03114-1. 29445156 PMC5812995

[ref57] Babu K. R.; Deepa M.; Nair C. M. K.; Vaidyan V. K. Growth of Anthraquinone Crystals by Gel Aided Solution Technique and Their Characterization. Bull. Mater. Sci. 1998, 21, 121–126. 10.1007/BF02927559.

[ref58] Latef A.; Bernede J. C.; Benhida S. Characterization of 9,10 Anthraquinone Thin Films. Thin Solid Films 1991, 195, 289–300. 10.1016/0040-6090(91)90280-B.

[ref59] Zhu L.; Liu J.; Liu Z.; Xie L.; Cao X. Anthraquinones with Ionizable Sodium Sulfonate Groups as Renewable Cathode Materials for Sodium-Ion Batteries. ChemElectroChem 2019, 6, 787–792. 10.1002/celc.201801252.

[ref60] Guo C.; Zhang K.; Zhao Q.; Pei L.; Chen J. High-Performance Sodium Batteries with the 9,10-Anthraquinone/CMK-3 Cathode and an Ether-Based Electrolyte. Chem. Commun. 2015, 51, 10244–10247. 10.1039/C5CC02251G. 26022356

[ref61] Larkin P. J. Infrared and Raman Spectroscopy: Principles and Spectral Interpretation, 2nd ed.; Elsevier: Oxford, New York, 2018.

[ref62] van Enckevort W. J. P.; Noorduin W. L.; Graswinckel S.; Verwer P.; Vlieg E. Epitaxy of Anthraquinone on (100) NaCl: A Quantitative Approach. Cryst. Growth Des. 2018, 18, 5099–5107. 10.1021/acs.cgd.8b00546. PMC6150655 30258306

[ref63] Lee M.; Hong J.; Kim H.; Lim H.-D.; Cho S. B.; Kang K.; Park C. B. Organic Nanohybrids for Fast and Sustainable Energy Storage. Adv. Mater. 2014, 26, 2558–2565. 10.1002/adma.201305005. 24488928

[ref64] Wielend D.; Vera-Hidalgo M.; Seelajaroen H.; Sariciftci N. S.; Pérez E. M.; Whang D. R. Mechanically Interlocked Carbon Nanotubes as a Stable Electrocatalytic Platform for Oxygen Reduction. ACS Appl. Mater. Interfaces 2020, 32615–32621. 10.1021/acsami.0c06516. 32573248 PMC7383929

[ref65] Silberstein K. E.; Pastore J. P.; Zhou W.; Potash R. A.; Hernández-Burgos K.; Lobkovsky E. B.; Abruña H. D. Electrochemical Lithiation-Induced Polymorphism of Anthraquinone Derivatives Observed by Operando X-Ray Diffraction. Phys. Chem. Chem. Phys. 2015, 17, 27665–27671. 10.1039/C5CP04201A. 26427626

[ref66] Hofer T. S.; Hünenberger P. H. Absolute Proton Hydration Free Energy, Surface Potential of Water, and Redox Potential of the Hydrogen Electrode from First Principles: QM/MM MD Free-Energy Simulations of Sodium and Potassium Hydration. J. Chem. Phys. 2018, 148, 222814 10.1063/1.5000799. 29907057

[ref67] Prasetyo N.; Hünenberger P. H.; Hofer T. S. Single-Ion Thermodynamics from First Principles: Calculation of the Absolute Hydration Free Energy and Single-Electrode Potential of Aqueous Li+ Using Ab Initio Quantum Mechanical/Molecular Mechanical Molecular Dynamics Simulations. J. Chem. Theory Comput. 2018, 14, 6443–6459. 10.1021/acs.jctc.8b00729. 30284829

[ref68] Prasetyo N.; Hofer T. S. Structure, Dynamics, and Hydration Free Energy of Carbon Dioxide in Aqueous Solution: A Quantum Mechanical/Molecular Mechanics Molecular Dynamics Thermodynamic Integration (QM/MM MD TI) Simulation Study. J. Chem. Theory Comput. 2018, 14, 6472–6483. 10.1021/acs.jctc.8b00557. 30336013

[ref69] Single-Ion Solvation; Theoretical and Computational Chemistry Series; Royal Society of Chemistry: Cambridge, 2011.

[ref70] Heeger A. J.; Sariciftci N. S.; Namdas E. B. Semiconducting and Metallic Polymers; Oxford University Press: New York, 2010.

[ref71] Muench S.; Wild A.; Friebe C.; Häupler B.; Janoschka T.; Schubert U. S. Polymer-Based Organic Batteries. Chem. Rev. 2016, 116, 9438–9484. 10.1021/acs.chemrev.6b00070. 27479607

[ref72] Oyaizu K.; Nishide H. Radical Polymers for Organic Electronic Devices: A Radical Departure from Conjugated Polymers?. Adv. Mater. 2009, 21, 2339–2344. 10.1002/adma.200803554.

[ref73] Nishide H.; Iwasa S.; Pu Y.-J.; Suga T.; Nakahara K.; Satoh M. Organic Radical Battery: Nitroxide Polymers as a Cathode-Active Material. Electrochim. Acta 2004, 50, 827–831. 10.1016/j.electacta.2004.02.052.

[ref74] Nakahara K.; Oyaizu K.; Nishide H. Organic Radical Battery Approaching Practical Use. Chem. Lett. 2011, 40, 222–227. 10.1246/cl.2011.222.

[ref75] Vlad A.; Arnould K.; Ernould B.; Sieuw L.; Rolland J.; Gohy J.-F. Exploring the Potential of Polymer Battery Cathodes with Electrically Conductive Molecular Backbone. J. Mater. Chem. A 2015, 3, 11189–11193. 10.1039/C5TA01500F.

[ref76] Clausen C.; Dražević E.; Andersen A. S.; Henriksen M. L.; Hinge M.; Bentien A. Anthraquinone Oligomers as Anode-Active Material in Rechargeable Nickel/Oligomer Batteries with Aqueous Electrolyte. ACS Appl. Energy Mater. 2018, 1, 243–248. 10.1021/acsaem.7b00009.

