# Supplementary material for: Analysis of the Ordering Effects in Anthraquinone Thin Films and Its Potential Application for Sodium Ion Batteries
Source: J Phys Chem C Nanomater Interfaces. 2021 Feb 10;125(7):3745–57. doi: 10.1021/acs.jpcc.0c10778 (PMC8016091; doi:10.1021/acs.jpcc.0c10778)
Supplement: Supplementary file 1 — jp0c10778_si_001.pdf [file jp0c10778_si_001.pdf]

Supporting Information:
Analysis of the Ordering Effects in Anthraquinone Thin Films and its Potential Application for Sodium Ion Batteries

Daniel Werner, [a] *Dogukan H. Apaydin,*[b] *Dominik Wielend,*[c] *Katharina Geistlinger,*[d] *Wahyu D. Saputri,*[e,f] *Ulrich J. Griesser,*[g] *Emil Dražević,*[h] *Thomas S. Hofer,*[i] * *and Engelbert Portenkirchner,*[a] *

[a] Institute of Physical Chemistry, University of Innsbruck, 6020 Innsbruck, Austria

[b] Institute of Materials Chemistry, TU Wien, 1060 Vienna, Austria

[c] Linz Institute for Organic Solar Cell (LIOS), Institute of Physical Chemistry, Johannes Kepler University Linz, 4040 Linz, Austria

[d] Institut für Ionenphysik und Angewandte Physik, Universität Innsbruck, 6020 Innsbruck, Austria

[e] Austrian-Indonesian Centre (AIC) for Computational Chemistry, Universitas Gadjah Mada, Sekip Utara, Yogyakarta 55281, Indonesia

[f] Indonesian Institute of Sciences, Sasana Widya Sarwono (SWS), 12710 Jakarta, Indonesia

[g] Institute of Pharmacy, University of Innsbruck, A-6020 Innsbruck, Austria

[h] Department of Engineering, Aarhus University, 8200 Aarhus N, Denmark

[i] Theoretical Chemistry Division, Institute for General, Inorganic and Theoretical Chemistry, University of Innsbruck, 6020 Innsbruck, Austria

Corresponding Author

*main corresponding author: engelbert.portenkirchner@uibk.ac.at, Phone: +43-512-507-58014

*corresponding author (theory): t.hofer@uibk.ac.at

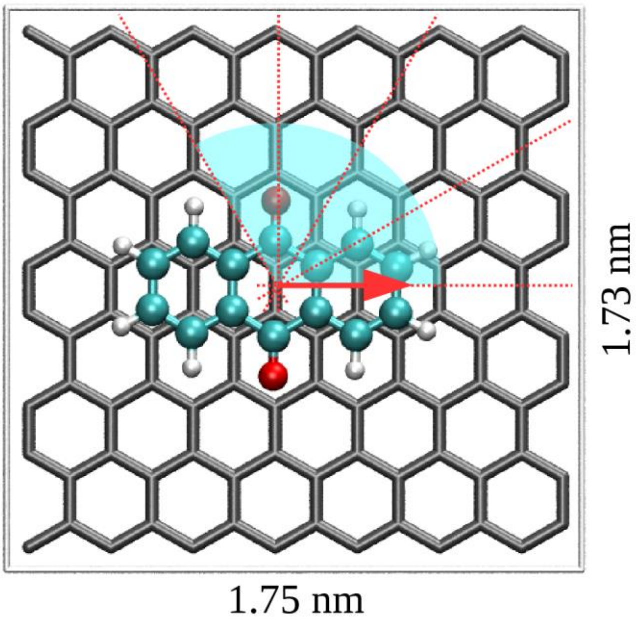

Figure S1. Unit cell of the surface model and sketch of the region employed to generate starting configurations for the conformation search via basing hopping. A total of 85 individual starting structures per substrate (i.e. AQ, AQ-Na and AQ-Na_2) have been generated considering 13 angular increments in the range [0°, 120°] (light blue region) along with five increments along the *a*-axis in the increments of [0, a_{Graph} /2] with a_{Graph} being the lattice constant of a single graphite unit of 0.246 nm.

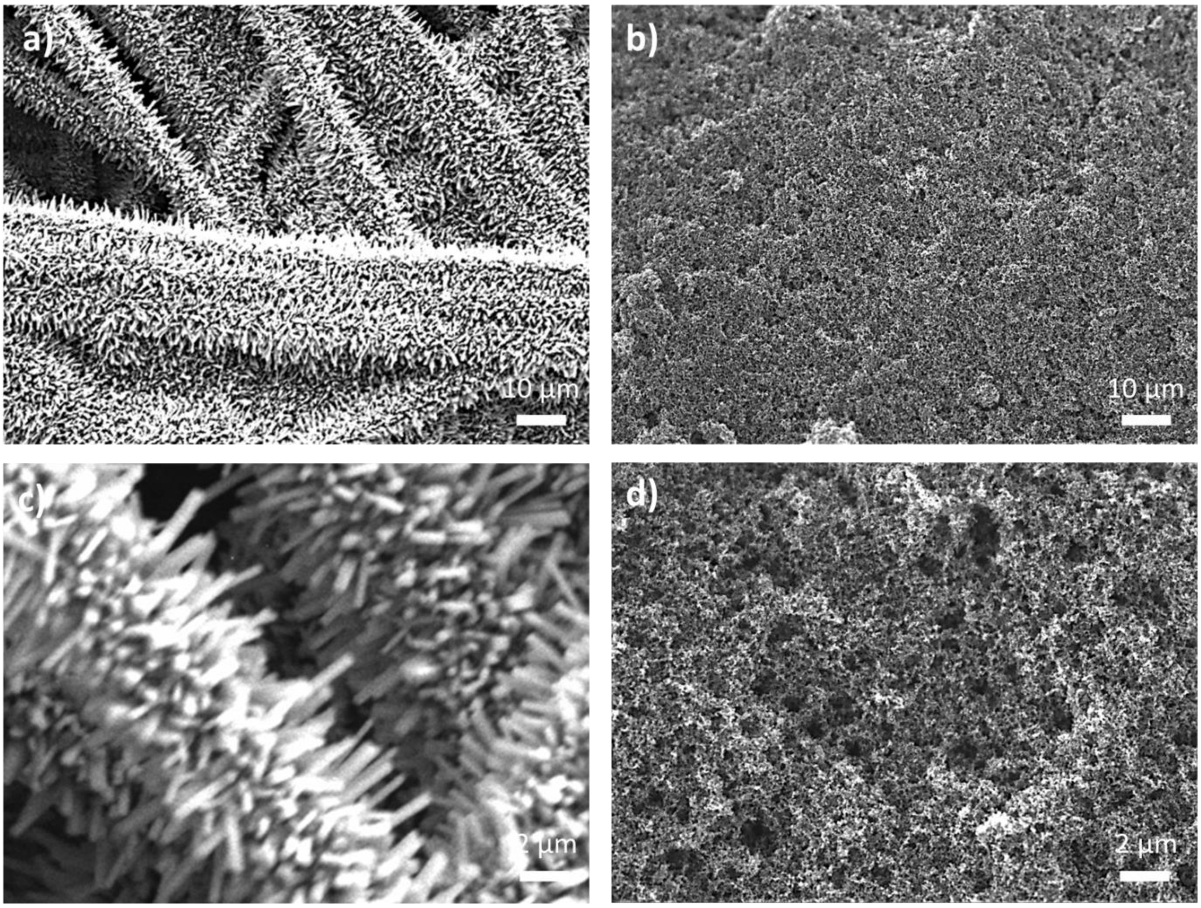

Figure S2. Scanning electron micrographs (SEM) of AQ thin film (a,c) and AQ powder (b,d) electrodes.

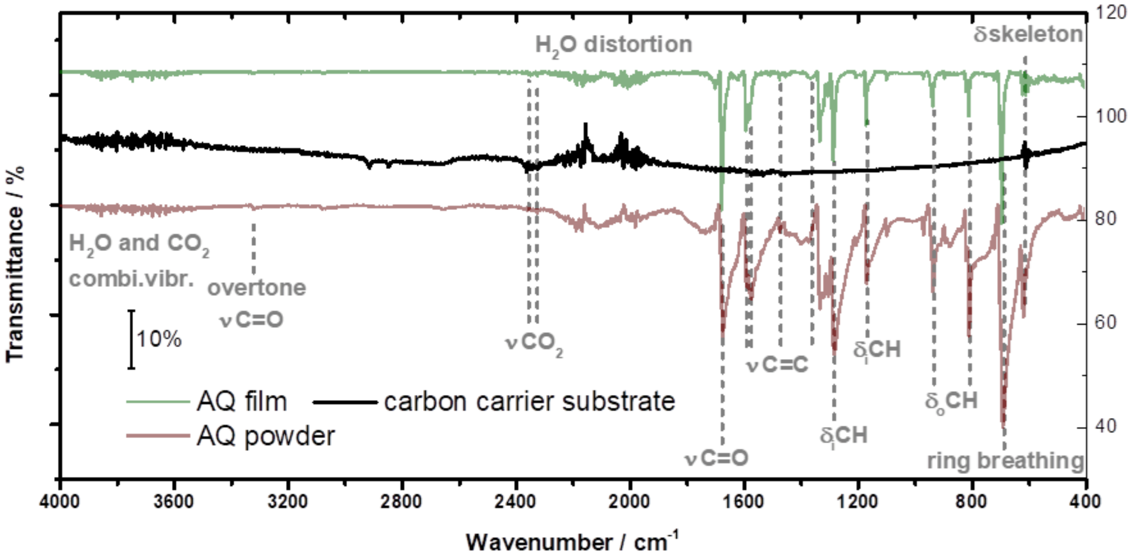

Figure S3. ATR-FTIR spectra of AQ thin film (green line), AQ powder (red line) and pure carbon carrier substrate (black line) electrodes in the frequency range from 4000 to 400 cm^{-1}.

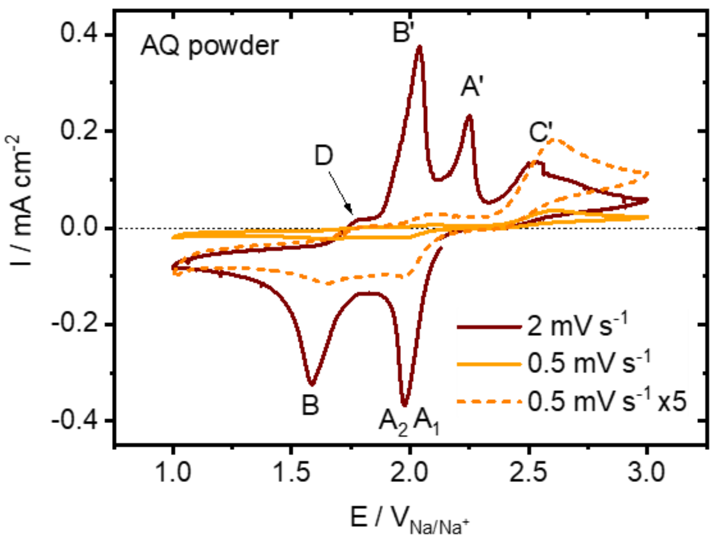

Figure S4. Cyclic voltammograms (CV) of AQ powder electrodes at scan rates of 2 and 0.5 mV s^{-1}. The voltammogram at 0.5 mV s^{-1} is also depicted with a fivefold magnification (dashed orange line) for better visibility.

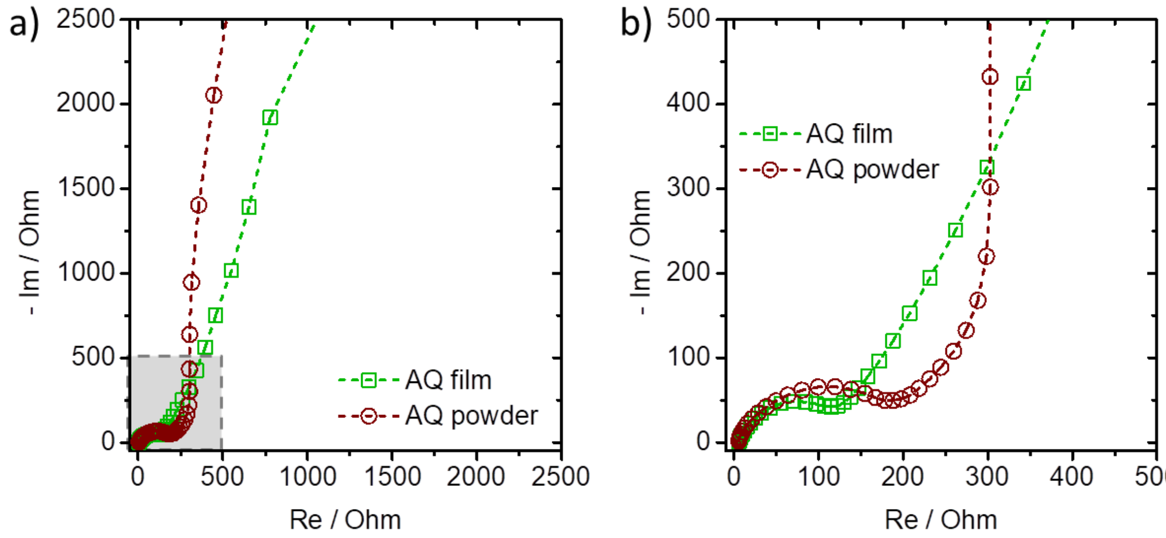

Figure S5. Nyquist plots of AQ thin film (green line) and AQ powder (red line) electrodes, recorded from 100 kHz to 10 mHz. A magnified view from 0 to 500 Ohm, highlighted by the grey shaded area in a), is shown in b). Dashed lines are a guide for the eye only and have no physical meaning.

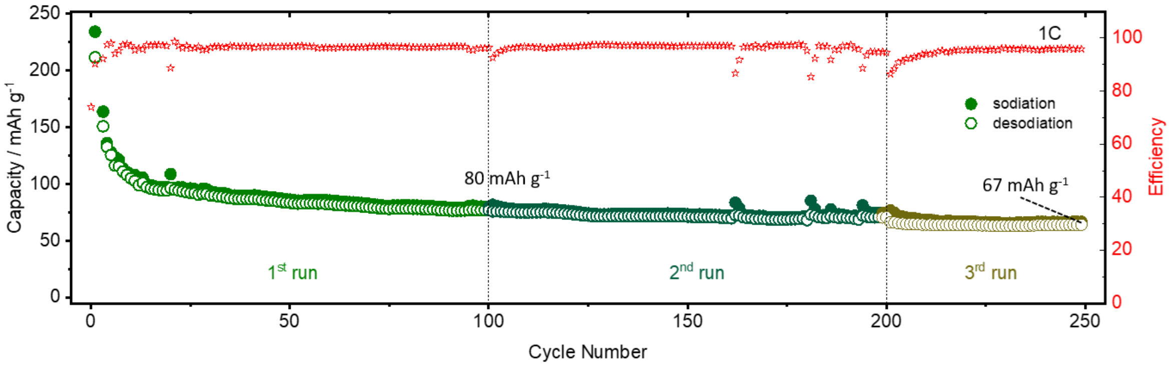

Figure S6. Long-term specific capacity (charge, open circles and discharge, closed circles) and the corresponding calculated efficiency versus cycle number (red stars), over 250 sodiation/desodiation cycles for AQ thin film electrodes, at a 1 C rate from 3.0 V to 1.0 V.

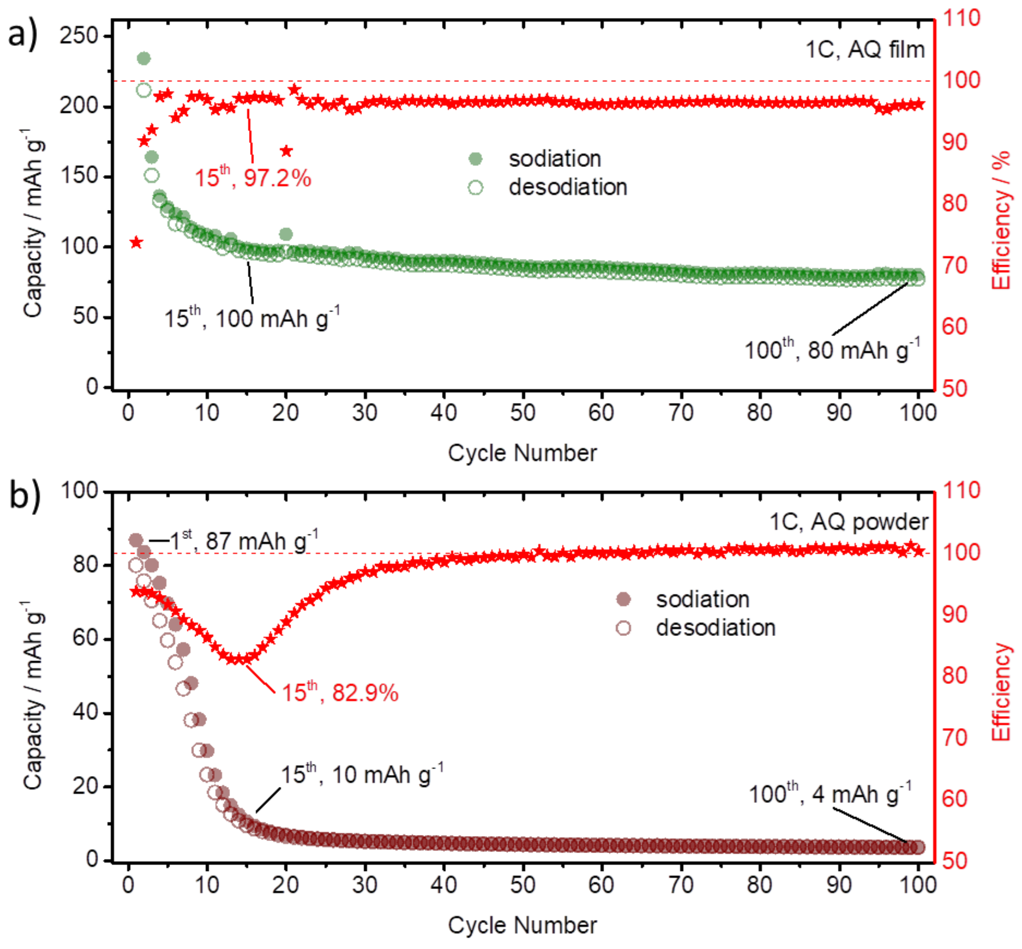

Figure S7. Comparison of the long-term specific capacity (charge, open circles and discharge, closed circles) and the corresponding calculated efficiency versus cycle number (red stars), over 100 sodiation/desodiation cycles for a) AQ thin film and b) AQ powder electrodes, at a 1 C rate from 3.0 V to 1.0 V.

Section S1. DFTB vs DFT

In order to validate the data obtained in this study, DFT/TZVP calculations of AQ on graphene employing the functionals PBESOL, B3LYP, HSESOL and LC-wPBESOL have been carried out. Since DFT is orders of magnitudes more demanding than its DFTB counterpart, only a single representative configuration (i.e. the ideal structural motif labeled Conf. 1 in **Figure 5a**, main text) was considered. Even with this limitation only a treatment considering a single layer of the carbon carrier (i.e. a graphene sheet consisting of 112 carbon atoms) proved feasible within the limits of the available computational equipment.

Table S1 compares the interaction energy U_{Int} and the average atom-surface distances for C-, H- and O-atoms of the AQ molecules obtained at different levels of theory. The interaction energy of -113.0 kJ mol^{-1} resulting from the SCC DFTB/3ob calculation compares well to the values obtained at DFT level being in the range of -107.1 to -140.4 kJ mol^{-1}, respectively. The best agreement is achieved with respect to the B3LYP functional. Similarly, the individual atom-surface distances of the DFTB calculation are in good agreement with those obtained via B3LYP and LC-wPBESOL, respectively.

Table S1. Interaction energy U_{Int} in kJ mol^{-1} and average atom-surface distance for C, H and O atoms in nm obtained for anthraquinone on graphene at different levels of theory.

**Method**	U_{Int} / kJ mol^{-1}	$z_{Surf\text{-}C}$ / nm	$z_{Surf\text{-}H}$ / nm	$z_{Surf\text{-}O}$ / nm
**SCC DFTB**	-113.0	0.307	0.308	0.300
**B3LYP**	-107.1	0.316	0.314	0.311
**LC-wPBESOL**	-130.8	0.305	0.304	0.296
**HSESOL**	-135.8	0.302	0.299	0.298
**PBESOL**	-140.4	0.300	0.297	0.295

It was also attempted to carry out a similar estimation of the interaction energy for the oxidized forms AQ-Na and AQ-Na_2. However, due to the ionic character of these systems, the calculation of the reference energies for the isolated molecular species (U_{mol}) proved to be much more intricate. Aside from convergence problems in the self-consistent field calculations the localization of a minimum structure on the potential energy surface proved unsuccessful. It should be noted that no such problems were encountered in the the SCC DFTB case, which is a result of the short-ranged nature of the associated energy, overlap and repulsive contributions in this approach.
